# Immunoproteasome and Non-Covalent Inhibition: Exploration by Advanced Molecular Dynamics and Docking Methods

**DOI:** 10.3390/molecules26134046

**Published:** 2021-07-02

**Authors:** Giulia Culletta, Maria Zappalà, Roberta Ettari, Anna Maria Almerico, Marco Tutone

**Affiliations:** 1 Dipartimento di Scienze e Tecnologie Biologiche Chimiche e Farmaceutiche (STEBICEF), Università degli Studi di Palermo, Via Archirafi 32, 90123 Palermo, Italy; giulia.culletta@unime.it (G.C.); annamaria.almerico@unipa.it (A.M.A.); 2 Dipartimento di Scienze Chimiche, Biologiche, Farmaceutiche ed Ambientali, Università di Messina, Viale Annunziata, 98168 Messina, Italy; maria.zappala@unime.it (M.Z.); roberta.ettari@unime.it (R.E.)

**Keywords:** immunoproteasome, non-covalent inhibitors, molecular dynamics, MD binding, metadynamics, induced-fit docking

## Abstract

The selective inhibition of immunoproteasome is a valuable strategy to treat autoimmune, inflammatory diseases, and hematologic malignancies. Recently, a new series of amide derivatives as non-covalent inhibitors of the β1i subunit with *K*_i_ values in the low/submicromolar ranges have been identified. Here, we investigated the binding mechanism of the most potent and selective inhibitor, *N*-benzyl-2-(2-oxopyridin-1(2H)-yl)propanamide (**1**), to elucidate the steps from the ligand entrance into the binding pocket to the ligand-induced conformational changes. We carried out a total of 400 ns of MD-binding analyses, followed by 200 ns of plain MD. The trajectories clustering allowed identifying three representative poses evidencing new key interactions with Phe31 and Lys33 together in a flipped orientation of a representative pose. Further, Binding Pose MetaDynamics (BPMD) studies were performed to evaluate the binding stability, comparing **1** with four other inhibitors of the β1i subunit: *N*-benzyl-2-(2-oxopyridin-1(2H)-yl)acetamide (**2**), *N*-cyclohexyl-3-(2-oxopyridin-1(2H)-yl)propenamide (**3**), *N*-butyl-3-(2-oxopyridin-1(2H)-yl)propanamide (**4**), and (*S*)-2-(2-oxopyridin-1(2H)-yl)-*N*,4-diphenylbutanamide (**5**). The obtained results in terms of free binding energy were consistent with the experimental values of inhibition, confirming **1** as a lead compound of this series. The adopted methods provided a full dynamic description of the binding events, and the information obtained could be exploited for the rational design of new and more active inhibitors.

## 1. Introduction

Protein turnover is essential for cellular function and homeostasis; in eukaryotic cells, the ubiquitin-proteasome system (UPS) is the central non-lysosomal pathway devoted to protein degradation. Whereas the lysosomal pathway mainly degrades membrane proteins or extracellular proteins imported into the cell by endocytosis, UPS, present both in the cytoplasm and nucleus, controls the degradation of damaged, incorrectly synthesized, or no longer useful intracellular proteins. Proteins are firstly tagged with several ubiquitin units; then, the polyubiquitinated proteins are rapidly hydrolyzed to small peptides by the proteasome, whereas ubiquitin is released and recycled [1]. The 26S constitutive proteasome consists of a barrel-shaped 20S catalytic core and two 19S regulatory caps. The catalytic core is constituted of four packed rings, each composed of seven different subunits, the two outer α, and the two inner β, respectively. The proteolytic activities reside in the β1c, β2c, and β5c subunits that are responsible for caspase-like (C-L), trypsin-like (T-L), and chymotrypsin-like (ChT-L) activities, respectively. 

**Figure 1:**
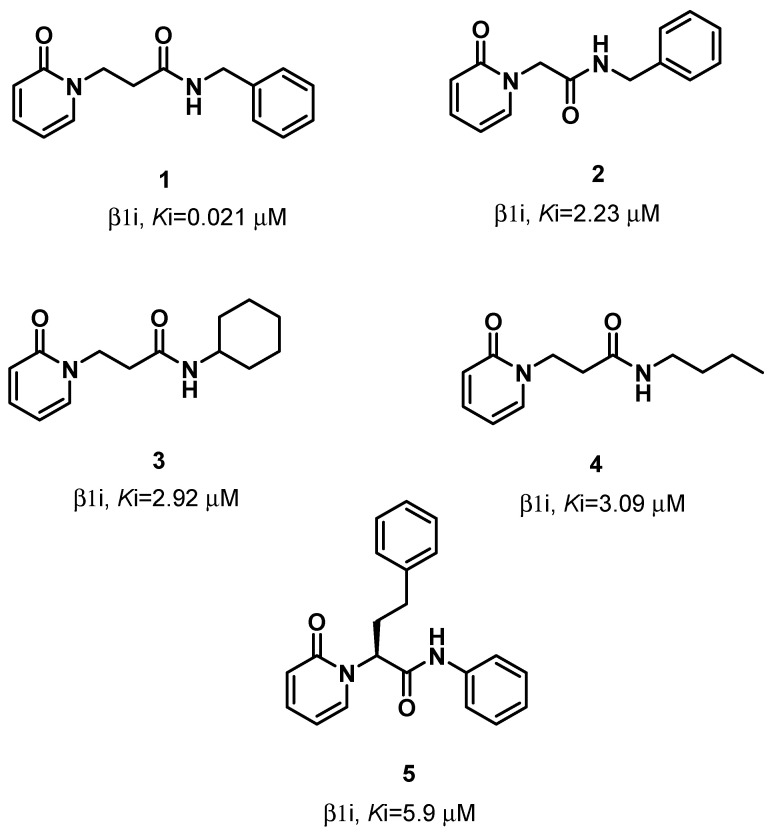
Structures and *K*_i_ values of the selective β1i inhibitors **1**–**5**.

Immunoproteasome is a specialized form of proteasome present in the vertebrates, constitutively expressed in lymphocytes and monocytes and induced by cytokines, such as IFN-α and TNF-α, in many other cell types. In immunoproteasomes, the constitutive catalytic subunits (β1c, β2c, and β5c) are replaced by the corresponding immunosubunits: β1i, β2i, and β5i. While β2i and β5i maintain the same type of activities as the β2c and β5c subunits, β1i mainly performs a ChT-L activity, thus cleaving peptides after hydrophobic amino acids [2]. High levels of immunoproteasomes have been found in a wide number of inflammatory diseases, such as Crohn’s disease or inflammatory bowel disease, and autoimmune diseases like rheumatoid arthritis or systemic lupus erythematosus [3]. Furthermore, immunoproteasomes are overexpressed in hematologic malignancies, including multiple myeloma or acute myeloid leukemia [4]. Therefore, the discovery of selective immunoproteasome inhibitors is pivotal to bring new chances for the treatment of the above-mentioned diseases. Exhaustive reports on selective covalent and non-covalent immunoproteasome inhibitors have been recently published [5,6]. The main class of covalent immunoproteasome inhibitors is that of peptide derivatives bearing an electrophile warhead able to interact with the nucleophilic hydroxyl group of catalytic Thr1. Just to give some examples, ONX-0914, a tripeptide α′,β′-epoxyketone, was the first β5i-selective inhibitor identified; another α′,β′-epoxyketone, UK-101, and the peptidyl aldehyde IPSI-001 showed a selective activity on the β1i subunit [5,6]. However, the covalent irreversible inhibition of a human enzyme is not always desirable in medicinal chemistry, as it can be responsible for potential toxicity due to off-target binding. Another drawback is that a single mutation in the catalytic amino acid (i.e., Thr1) could cause a loss of activity and acquired resistance. [7]. Non-covalent inhibition is therefore strongly desirable, because it is free of these disadvantages. Lacking a reactive warhead, non-covalent inhibitors may have an improved selectivity and less reactivity and instability and, therefore, may not exhibit the side effects that occur in covalent inhibitor therapies (e.g., liver toxicity and idiosyncratic adverse reactions) [8,9]. Furthermore, the enzyme–inhibitor complexes have reduced lifetimes, and this promotes an extensive tissue distribution of the drug [10]. To date, few non-covalent immunoproteasome inhibitors show selectivity towards the β1i and/or β5i subunits. One of them is Argyrin B, a natural cyclic peptide that is a reversible, noncompetitive inhibitor of β5i and β1i [8]. Other compounds are N,C-capped dipeptides, such as PKS2279 and PKS2252, in which the insertion of a β-amino acid markedly reduces the inhibitory potency against constitutive proteasomes, yet maintain potent inhibitory activity against immunoproteasomes [11]. Recently, some of us identified a panel of selective non-covalent inhibitors of the β1i and/or β5i subunits, characterized by a 2(1H)-pyridone scaffold linked to an amide function [12]. *N*-Benzyl-2-(2-oxopyridin-1(2H)-yl)propanamide (**1**) proved to be the most potent and selective inhibitor, with a *K*i = 21 nM against the β1i subunit. Four other compounds of this series, *N*-benzyl-2-(2-oxopyridin-1(2H)-yl)acetamide (**2**), *N*-cyclohexyl-3-(2-oxopyridin-1(2H)-yl)propanamide (**3**), *N*-butyl-3-(2-oxopyridin-1(2H)-yl)propanamide (**4**), and (*S*)-2-(2-oxopyridin-1(2H)-yl)-*N*,4-diphenylbutanamide (**5**), showed remarkable inhibitory activity towards the β1i subunit (Figure 1). Derivatives **3**–**5** were also active against the β5i subunit, whereas none of the compounds **1**–**5** proved to affect the constitutive catalytic subunits.

**Figure 2:**
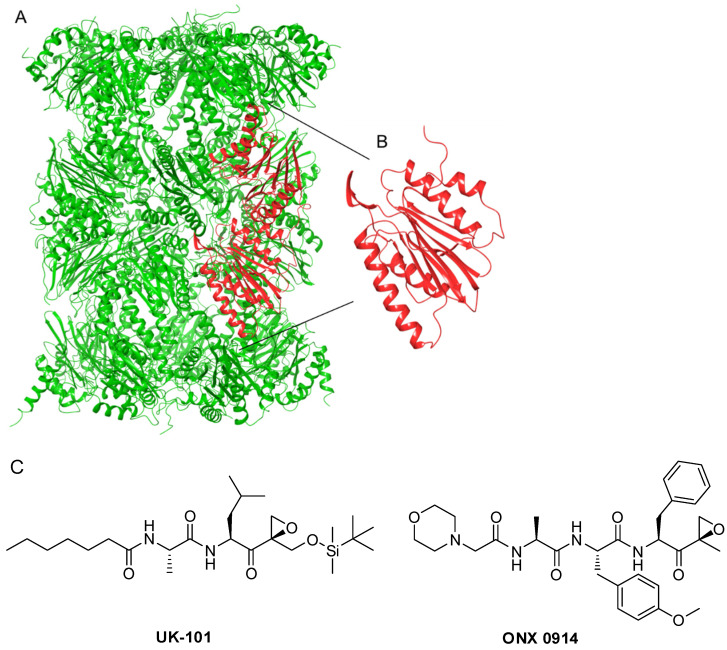
(**A**) 3D structure of the immunoproteasome, the two β1i subunits in red, (**B**) 3D structure of the β1i subunit, and (**C**) the structure of inhibitors UK-101 and ONX-0914.

The available experimental structures of immunoproteasomes provided the basis for several computational investigations. In the recent past, most of these studies made use of molecular docking methods. In particular, the binding mode of the non-covalent amide derivatives **1** and **2** was investigated at this level [12], while, to the best of our knowledge, the most accurate computational investigations were performed just on the β1i subunit (Figure 2A,B) and the peptide α′,β′-epoxyketone UK101 (Figure 2C) using molecular dynamics (MD) simulations. The observed selectivity of UK101 for the β1i subunit is rationalized by the requirement for both a linear hydrocarbon chain at the N-terminus and a bulky group at the C-terminus of the inhibitor [13]. In recent years, the constant update of hardware capabilities allowed the development of enhanced MD methods able to provide a full dynamical description of the target–ligand-binding events [14]. These methods are usually employed given that the sampling issue is fundamental to describing these slow processes while docking methods continue to be pivotal to screening large libraries, also assisted by MD [15,16].

In this manuscript, we investigated the binding mechanism of the previously identified most active non-covalent amide **1** in the β1i subunit. For this purpose, we employed advanced molecular dynamics methods, such as MD binding (MDB) [17] and Binding Pose MetaDynamics (BPMD) [18]. In particular, we used the MDB tools implemented in the BiKi suite [19] to analyze the binding mechanism and gain insights into the ligand entrance pathway. Then, plain MD was performed to study the stability and conformational space into the immunoproteasome–ligand complex, thus allowing elucidation of the compound dynamic behaviors within the binding cavity. Successively, a cluster analysis provided representative poses that were used to evaluate the binding stability using the BPMD protocol. To have a comparative point of view, we also carried out induced-fit docking (IFD) and BPMD studies of the other four compounds (**2**–**5**) that showed high inhibitory activities towards the β1i subunit. The results obtained could provide further information to develop the most selective and active immunoproteasome inhibitors.

## 2. Results

### 2.1. MD-Binding (MDB) Analysis

**Figure 3:**
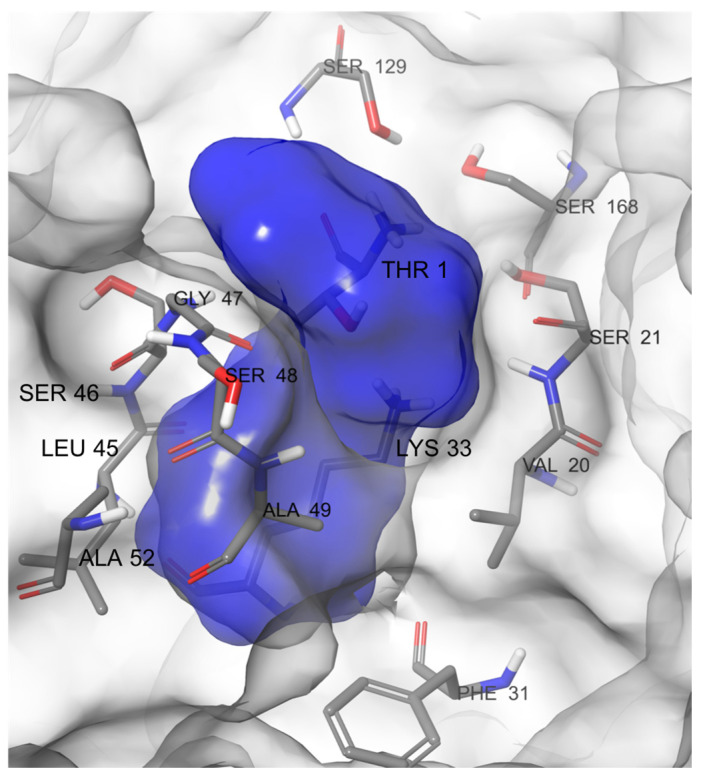
Identification of the binding cavity of the β1i subunit (solid blue) by NanoShaper software with the residues involved in the binding pocket.

We began the study using the crystal structure of the murine immunoproteasome in complex with the inhibitor ONX-0914 (Figure 2C) bound to the β5i subunit (PDB ID: 3UNF) [20]. Murine and human immunoproteasomes share a sequence identity of more than 90%, and the few nonidentical residues were external to the active sites. In the literature, a crystal structure of human immunoproteasome was recently released in complex with a boronic acid derivative [21], but the docking of compound 1 was previously studied on the β1i subunit structure derived from the PDB ID:3UNF that do not bind any ligand. For these reasons, we used it as a starting point to carry out our simulations. To gain insights into the ligand-binding mode, we employed the MDB method to predict the path of ligand entrance into the cavity. This method has the advantage of describing complex events without incurring prohibitive time and computational costs. It is based on an adaptive, electrostatics-inspired bias and a campaign of trivially parallel short MD simulations to identify a near-native binding pose from the sampled configurations. At a reasonable computational cost, this method also provides accurate predictions when the starting protein conformation is different from that of the crystal complex, a known hurdle for traditional molecular docking [22]. The advanced proposed MDB protocol would require the identification of the binding pocket with NanoShaper software [23], which can identify the atoms facing the lumen pocket entrance in the protein target. According to NanoShaper software, the attractive protein residues selected were Thr1, Val20, Ser21, Phe31, Lys33, Leu45, Ser46, Gly47, Ser48, Ala49, Ala52, Ser129, and Ser168 (Figure 3).

Compound **1** is positioned with a random orientation at a predetermined distance, measured in terms of the thickness of the solvation shell around the ligand. From the set, we started 20 replicas of 20 ns for each entrance starting from the apo structure, thus collecting a total of 400 ns of MDB simulations.

**Figure 4:**
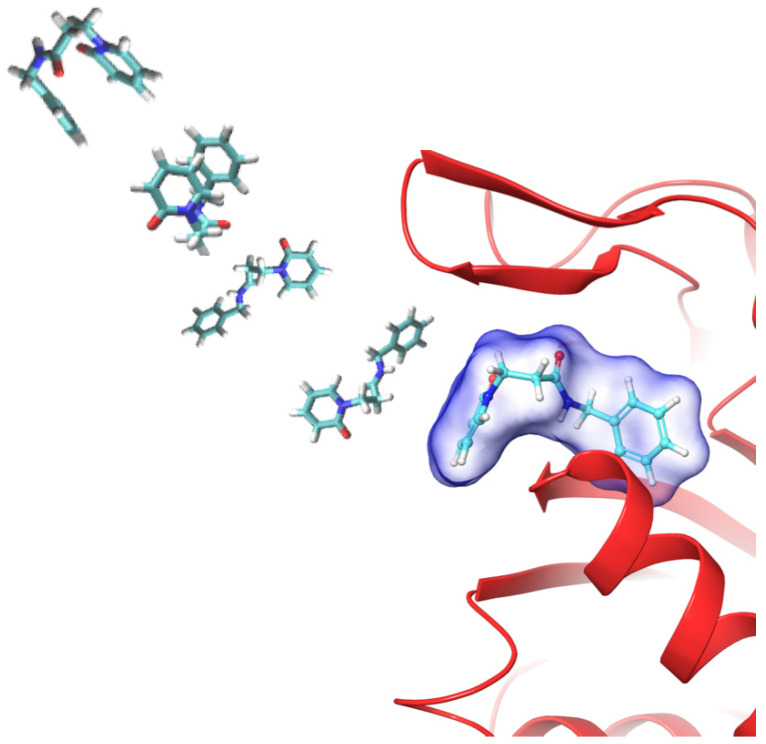
Time sequence of compound **1** approaching the active site of the β1i subunit studied by MD binding. The figure is representative of the 20 replicas.

The analysis of the results showed that the simulations overcome the energetic barrier in an average time of 2 ns, reaching the binding site. The unavailability of crystallographic structures for non-covalent ligand 1 did not allow the comparison of the conformations, employing the RMSD of the bound ligand. For these reasons, the RMSD of the protein backbone was used as a reference for any uncommon behaviors. All replicas showed a protein backbone RMSD average <2 Å, decreasing when the ligand arrived at the binding site (Supplementary Materials, Figures S1–S7). In most replicas, the ligand entered into the active site in the following 8 ns of the simulations, and in the last 10 ns, its refinement at the binding site was registered (Figure 4).

After the first 20 ns, the electrostatic bias was removed, and the sampling time was increased starting from the final frames of each MDB replica to enhance the sampling conformational changes and interactions of the ligand inside the binding site. For each replica, 10 ns more of the simulation was carried out, collecting a total of 600 ns of MD simulations. The plain MD simulations performed after the bias switch-off provided the local refinement of the binding mode. Once the binding simulation campaign was completed, the replicas ending without the ligand into the binding site were pruned out, and the remaining replicas were analyzed. The major part of the simulations showed a high stability, with the ligand strictly bound to the binding pocket, and in a few simulations, the ligand rapidly drifted away. Twelve replicas maintained a high stability at the binding site, as shown by averaged value of RMSD 1.5 Å of the complex (Supplementary Materials, Figures S8–S10). Then, the 12 replica trajectories were clustered. Each trajectory was recorded in 1000 frames, and these frames were clustered considering the RMSD of the binding site backbone (12,000 frames total). Each replica returned three representative clusters for a total of 36 MD representative poses. These last ones were further clustered and took into consideration the conformations of the ligand into the binding site and the most occurred interactions. In the end, it was possible to identify three final representative poses (pose 1, pose 2, and pose 3).

The poses obtained from the clustering analysis were characterized by the important features observed during the simulations. In particular, in pose 1, two H-bonds were formed between the oxygen of the amide group and Ser21 and between the hydrogen of the amide group and Gly47 (Supplementary Information, Figure S11A). The binding of the ligand was strengthened by several van der Waals contacts between the benzyl group and the residues Val20, Phe31, Lys33, Gly47, Ala49, and Ala52. Val20, Ser21, Ser46, Gly47, Ala49, and Ala52 interacted with the linker between the two rings.

The identified pose 2 showed a series of noteworthy interactions that have not been previously identified. The benzyl group of pose 2 interacted by a pi-stacking interaction with Phe31. This pose was stabilized by several van der Waals contacts. The 2-pyridone moiety showed a series of contacts different from pose 1 (Lys33, Leu45, Ser46, Gly47, Ser48, Ala49, Ala52, Ser129, and Ser168). Ser21, Phe31, Ser46, and Gly47 interacted with the ethylene linker. It is worthy to note the absence of H-bonds in this pose (Supplementary Information, Figure S11B).

Pose 3 was characterized by the same H-bonds network observed in pose 1, with Ser21 and Gly47 residues. The 2-pyridone moiety formed one cation-pi-stacking interaction with the epsilon amino group of Lys33 (Supplementary Information, Figure S12A). As observed for the other poses, van der Waals contacts strengthened the ligand binding in pose 3. The benzyl group interacted with Ser21, Ala22, Leu45, and Ser46. The 2-pyridone moiety showed contacts with several residues: Phe31, Lys33, Gly47, Ser48, Ala49, and andAla52. The linker showed interactions with Thr1, Ser46, Gly47, and Ser168. 

**Figure 5:**
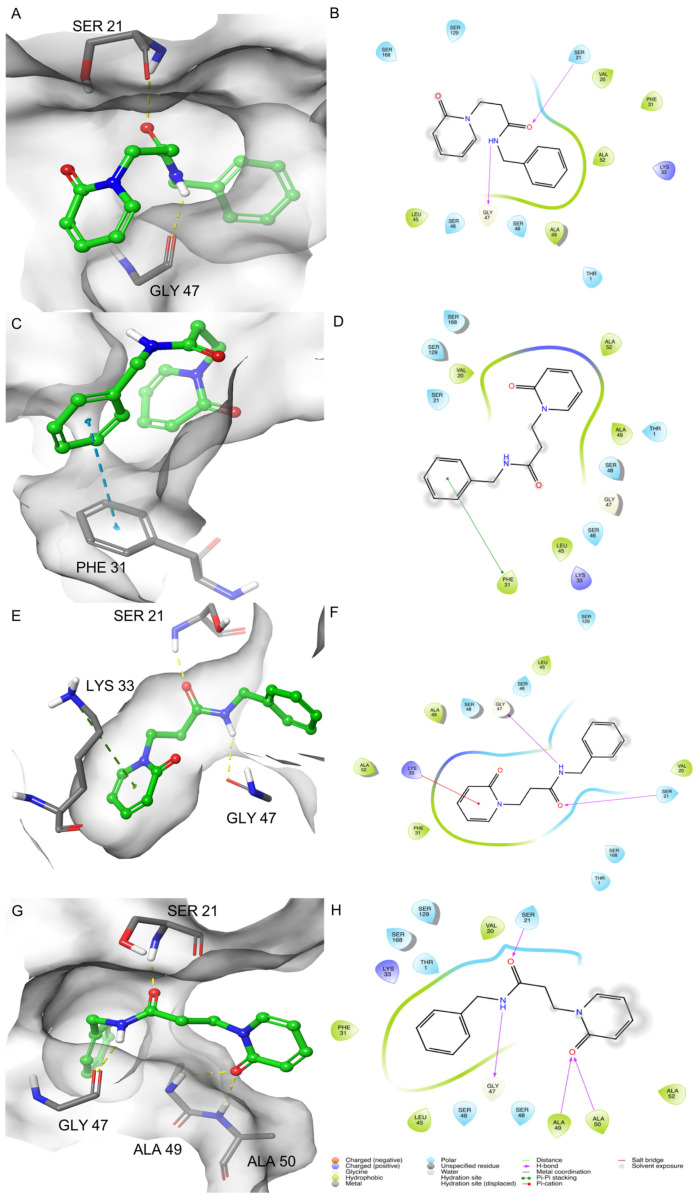
3D and 2D-binding modes of compound 1: pose 1 (**A**,**B**), pose 2 (**C**,**D**), and pose 3 (**E**,**F**) after the MDB simulations and after IFD (**G**,**H**) into the β1i active site of murine immunoproteasome (PDB ID: 3UNF). In the 3D figures, the H-bonds are represented in yellow dashes, the cation-pi-stacking interactions in green dashes, and the pi-pi stacking in blue dashes.

The major differences observed for these poses concerned the orientations of pose 1 and pose 3 related to their interactions with the residues of the binding site. In particular, besides the same H-bonds, a flipped orientation of the 2-pyridone and the benzyl moieties was observed. This evidence could reveal that the entrance mode of the ligand occurred in different ways without affecting the binding capability during the MD runs. The folded conformation assumed by the ligand in pose 2 seemed to represent an intermediate conformation. Concerning previous studies [12], two pi-stacking interactions and van der Waals contacts between the rings and the residues Thr1, Val20, Phe31, Lys33, Leu45, Ser46, and Ala52 were identified (Figure 5).

### 2.2. Induced Fit Docking (IFD)

To add more information concerning the previous docking studies and compare the results obtained by the MDB, we used the more accurate protocol induced-fit docking (IFD) [24]. IFD predicts the ligand-binding modes and concomitant structural changes in the receptor. It confers flexibility to the protein side chains, allowing the ligand to adjust and optimize the binding interactions within the active site. IFD was carried out for compound 1 and for the other four compounds **2**–**5** that showed encouraging inhibitory activity on β1i. In the previous studies, classical docking was performed for compound **2**, while computational studies have not been performed yet for compounds **3**–**5**. 

The best IFD pose of **1** reported the same two interactions observed after the MD simulation: the H-bonds between the residues Gly47 and Ser21 of the protein and NH amide and the carbonyl group of the molecule (Figure 5G,H). Besides, other hydrogen bonds were found. In particular, the carbonyl of 2-pyridone moiety formed an H-bond network with Ala49 and Ala50. Concerning the other MDB poses, this was a peculiar difference of the IFD pose that was not observed in the docking study. These residues, together with Ser48, stabilized the ligand binding by van der Waals contacts, such as observed in the previous docking study. Other van der Waals contacts were formed between the benzyl group and Val20, Phe31, Lys33, Leu45, Gly47, and Ala52. Finally, the ethylene linker between the rings interacted with Thr1, Val20, Ser21, Gly47, and Ala49. It is interesting to note that the pi-stacking interactions observed in pose 2 between the benzyl group and Phe31 and the cation-pi-stacking interaction in pose 3 between the 2-pyridone and Lys33 were not evidenced in the IFD pose but only as van der Waals contacts (Figure 5).

**Figure 6:**
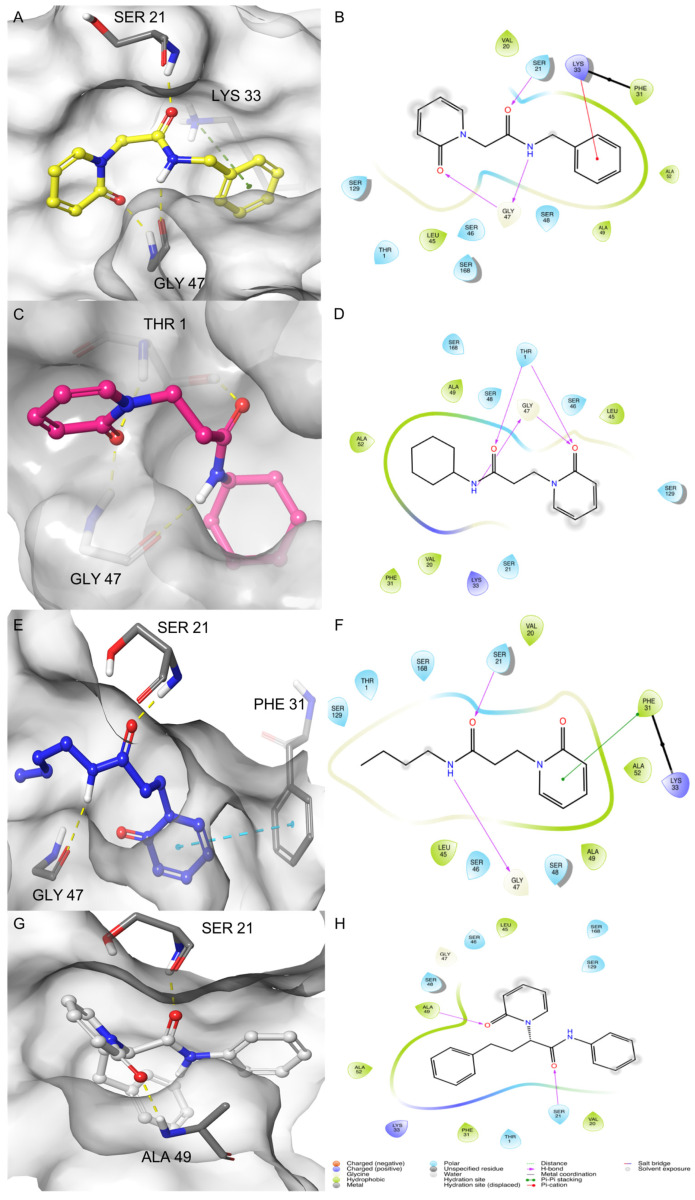
3D and 2D-binding modes of compound **2** (**A**,**B**), compound **3** (**C**,**D**), compound **4** (**E**,**F**), and compound **5** (**G**,**H**) into the β1i active site of murine immunoproteasome (PDB ID: 3UNF) after the IFD study. In the 3D figures, the H-bonds are represented in yellow dashes, the cation-pi-stacking interactions in green dashes, and the pi-pi stacking in blue dashes.

The other four analogs of amide 1 were characterized by structural variations at the *N*-substituent and the methylene/ethylene linker between the 2-pyridone scaffold and the amide function. Compound 2 showed a methylene linker between the 2-pyridone scaffold and the amide function, and the experimental activity was recorded with a *K*i value of 2.23 µM on the β1i subunit. The best IFD pose for 2 showed three H-bonds: Ser21 with the carbonyl of amide and Gly47 with the NH amide and the carbonyl of 2-pyridone. The benzyl moiety of the molecule formed a cation-pi-stacking interaction with Lys33, as also evidenced for 1 in pose 3 (Figure 6A,B).

The cyclohexyl derivative **3** (*K*i = 2.92 µM) formed four H-bonds. The residue Thr1 made two H-bonds with the carbonyl of amide and the carbonyl of 2-pyridone. Gly47 formed two H-bonds with NH amide and carbonyl of 2-pyridone (Figure 6C,D). The interactions of the best IFD pose of *n*-butyl derivative **4** (*K*i = 3.09 µM) were characterized by two H-bonds between the carbonyl and NH of the amide of the molecule with Ser21 and Gly47, respectively. The 2-pyridone moiety formed pi-pi stacking with the Phe31 (Figure 6E,F). The last compound, (*S*)-2-(2-oxopyridin-1(2H)-yl)-*N*,4-diphenylbutanamide (**5**) (*K*i = 5.9 µM), showed two H-bonds, one between Ser21 and carbonyl of amide and the other between Ala49 and carbonyl of 2-pyridone (Figure 6G,H). Additionally, for these molecules, the recurrent interactions were between the residues Ser21, Gly47, and the amide group, but it underlined the pi-stacking interactions with Phe31 and Lys33, which could constitute clear evidence of the key role of these residues in the inhibition pattern.

### 2.3. Binding Pose MetaDynamics Analysis 

Binding Pose MetaDynamics (BPMD) is an automated, enhanced sampling, metadynamics-based protocol, in which the ligand is forced to move around its binding pose. The possible higher mobility of the ligand under a biasing potential is a mark of the binding mode instability. This method showed the ability to reliably discriminate between the ligand-binding pose retrieved by MDB and a plausible alternative generated with IFD studies [22]. 

We decided to use BPMD to evaluate the affinity of the three representative poses obtained from MDB and the pose of IFD into the binding site for compounds **1** and **2**–**5**. The results were defined in terms of the pose stability based on the PoseScore, which is the RMSD of the ligand related to the starting coordinates of the heavy atoms of the ligand. Moreover, to evaluate the results, another metric is used, such as the PersScore, which is a clue of the H-bond formed between the ligand and the target during the simulations. The linear combination of these two scores provides a third score, the CompScore, which is calculated with Equation (1): 

(1) CompScore = PoseScore − 5 × PersScore

Lower values of the CompScore indicate more stable complexes. 

**Figure 7:**
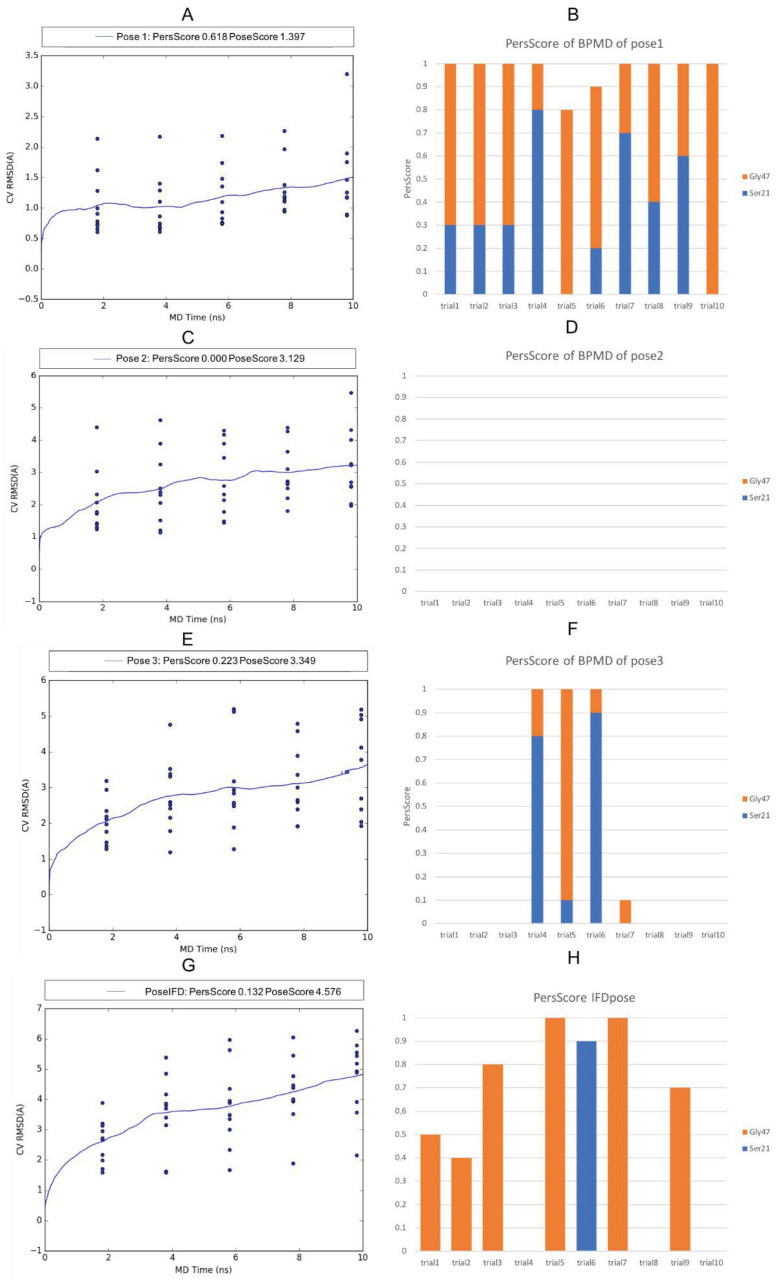
Plots of the RMSD estimate averaged over all 10 trials vs. the simulation time for the BindingPoseMetaDynamics runs: pose 1 (**A**), pose 2 (**C**), pose 3 (**E**), and IFD pose (**G**). Persistence Score Plots: pose 1 (**B**) pose 2 (**D**), pose 3 (**F**), and IFD pose (**H**). The blue dots represent the values of the CV RMSD at different times (2 ns, 4 ns, 6 ns, 8 ns, and 10 ns) for each simulation trial. The blue lines represent the mean CV RMSD values along the 10 × 10 ns of the simulation trials. The orange and blue bars represent the fraction of H-bonds maintained during the simulation for each trial.

During the metadynamics simulations, pose 1 reached a steady PoseScore = 1.397, considered stable, while the PersScore showed that the hydrogen bonds identified at the start of the metadynamics run were kept for 60% of the averaged time (Figure 7A). In particular, the H-bond between the NH amide group of the ligand and Gly47 was kept for 88% of the simulation time, while the H-bond between the carbonyl of the ligand and Ser21 for 36% (Figure 7B). The CompScore value was -1.694. Due to the absence of H-bonds recorded, pose 2 with recorded pi stacking and van der Waals interactions showed the same value for the PoseScore and CompScore, 3.129 (Figure 7C), while, for pose 3, the scores were PoseScore = 3.349, PersScore = 0.223, and CompScore = 2.235, respectively (Figure 7E). As for pose 1, pose 3 kept the H-bond between NH amide and Gly47 as 26% and 18% between carbonyl and Ser21 (Figure 7F). 

The PoseScore for the pose of amide 1 obtained by the IFD was 4.576, and the PersScore showed that the hydrogen bonds identified at the start of the MetaDynamics run were kept for 13% of the averaged time. The value of the CompScore was 3.917 (Figure 7G). It is interesting to point out that, of the four H-bonds detected by IFD, the two interactions between the amide group and Ser21 and Gly47 were maintained—in particular, the interaction between NH amide and Gly47 for 43% and 9% between carbonyl and Ser21 (Figure 7H). 

The RMSD values and the percentage of the H-bonds retrieved from BPMD studies for the amide 1 in the three MDB poses and in the IFD pose showed that pose 1 could be considered more stable. Pose 1, pose 3, and the IFD pose adopted the same plain conformation and H-bonds between Ser21, Gly47, and the amide group. The differences were in the additional interactions between Ala49, Ala50, and the carbonyl of 2-pyridone, which led to a rotation of 2-pyridone, causing the ring to be specular in the IFD pose and showed a high value of RMSD (4.02 Å).

**Figure 8:**
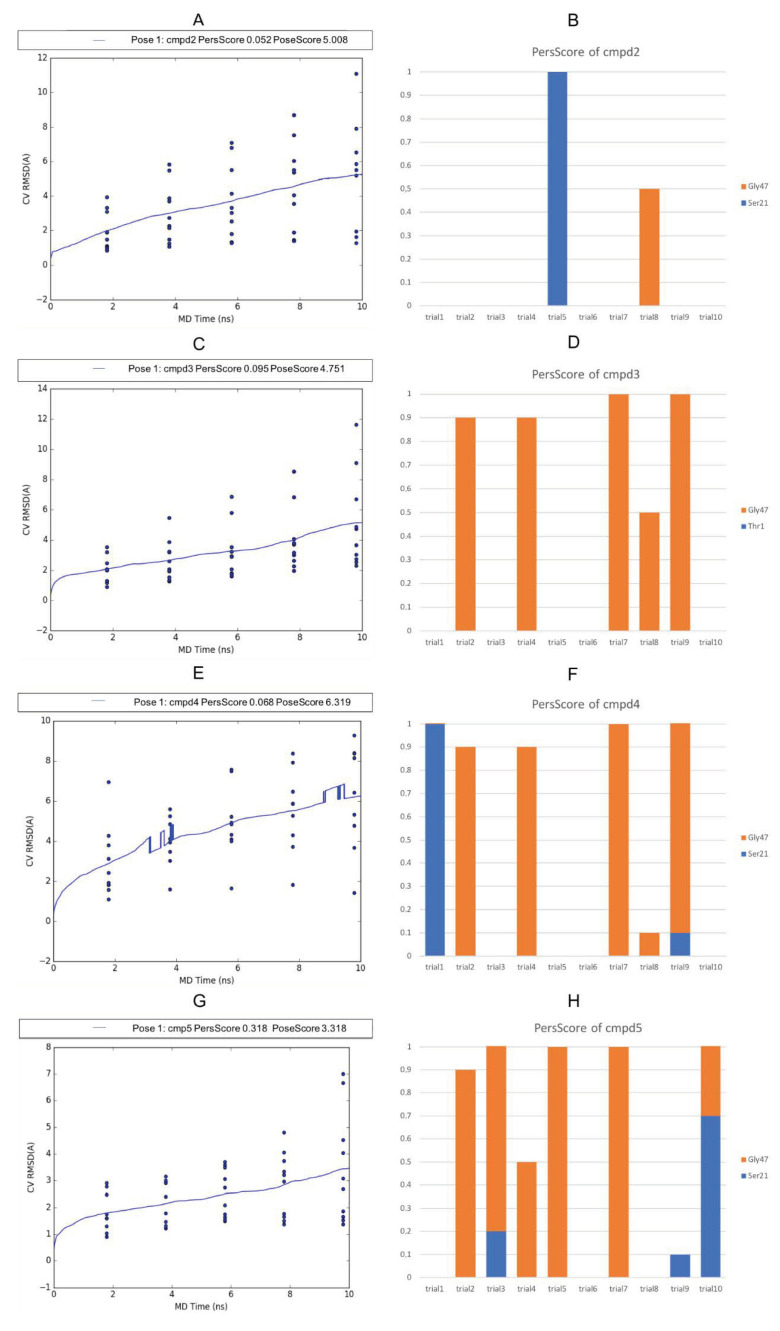
Plots of the RMSD estimate averaged over all 10 trials vs. the simulation time for the BindingPoseMetaDynamics runs: compound **2** (**A**), compound **3** (**C**), compound **4** (**E**), and compound **5** (**G**). Persistence Score plots of compounds **2** (**B**), compound **3** (**D**), compound **4** (**F**), and compound **5** (**H**). The blue dots represent the values of the CV RMSD at different times (2 ns, 4 ns, 6 ns, 8 ns, and 10 ns) for each simulation trial. The blue lines represent the mean CV RMSD values along the 10 × 10 ns of the simulation trials. The orange and blue bars represent the fraction of H-bonds maintained during the simulation for each trial.

**Figure 9:**
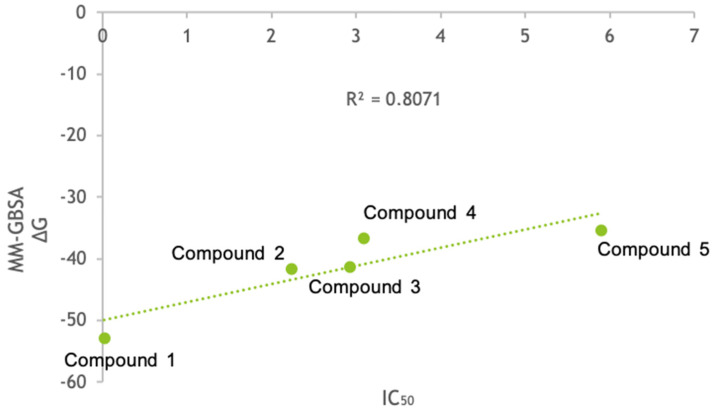
The plot of the MM-GBSA ∆G binding vs. the *K*i values of compounds **1**–**5**. The binding free energy is expressed in Kcal/mol, and the IC_50_ is expressed in µM.

The BPMD analysis was also carried out for compounds **2–5** to evaluate their binding stability with respect to the most active compound of the series, **1**. The results of the BPMD calculations are reported in Figure 8. As can be evidenced from the plots, all showed PoseScore values higher than the averaged PoseScore for **1**. The hydrogen bonds identified at the start of the MetaDynamics run were maintained for 10-30% of the averaged time (Figure 8B,D,F,H) The CompScore values for compounds **2**–**5** were 4.750, 4.276, 5.979, and 1.728, respectively. Moreover, MM-GBSA-binding free energy calculations for all the complexes were performed. The plot of the calculated ∆G binding vs. the *K*i values is reported in Figure 9, and it shows an R^2^ = 0.8071 (compound **1** ∆G = −52.912 Kcal/mol, compound **2** ∆G = −41.684 Kcal/mol, compound **3** ∆G = −41.355 Kcal/mol, compound **4** ∆G = −36.701 Kcal/mol, and compound **5** ∆G = −35.340 Kcal/mol). 

## 3. Discussion

The inhibition of the human immunoproteasome is a hot topic of recent years in medicinal chemistry due to its involvement in a wide range of diseases. Promising immunoproteasome inhibitors, both covalent and non-covalent, have been recently identified. In covalent inhibitors, the presence of a reactive warhead may cause significant off-target activities against other proteins, which may result in side effects (e.g., liver toxicity and idiosyncratic adverse reactions) and reduced selectivity over time [8,9]. For these reasons, the attention was focused on non-covalent immunoproteasome inhibitors. In this context, a series of amide derivative β1i subunit inhibitors with *K*_i_ values in the low micromolar or submicromolar range have been recently identified [12]. The use of computational approaches could characterize the binding process of these inhibitors—in particular, the use of advanced molecular dynamics approaches able to explore the dynamic features of the protein/ligand complex could overcome the limitations of semiflexible molecular docking methods in which the protein target is treated as a rigid body. Several advanced methods have been proposed in the last years for computing association and dissociation mechanisms, and all of them were shown to be promising in the interpretation of such mechanisms. With the aim to gain more insights into non-covalent inhibitors of the immunoproteasome, we decided to exploit these enhanced sampling methods. 

Here, we investigated the dynamic binding mechanism of compound **1**, the most active of a series of non-covalent amide derivatives. With the aim of collecting mechanistic insight on the binding process, we performed the MDB protocol implemented in BiKi software to simulate the events that elapsed among the ligand unbound and the ligand entrance in the binding pocket. Successively, plain MD simulations were performed to extend the sampling of the bound states. The clustering of the survived complexes trajectories allowed identifying three representative poses (pose 1, pose 2, and pose 3) observed during the simulation. The most important interactions for the inhibition pattern were, in pose 1, two H-bonds between the amide group and Ser21 and Gly47 and, in pose 2, the benzyl group interacting by pi-pi stacking with Phe31. The residues Ser21 and Gly47 of pose 3 formed H-bonds with carbonyl and NH amide, and at the same time, the 2-pyridone moiety made a cation-pi-stacking interaction with the epsilon amino group of Lys33. Moreover, pose 1 showed a different orientation of the 2-pyridone moiety with respect to the docking and IFD studies. The 2-pyridone moiety was stabilized in the binding pocket by van der Waals contacts, as observed in MDB, while, in docking and IFD studies, it was stabilized by H-bonds with Ala49 and Ala50 beyond van der Waals contacts. In pose 3, the peculiarity is represented by the 2-pyridone moiety interacting with the pi-stacking interaction with Lys33, which determined a flipped orientation with respect to pose 1. Finally, in pose 2, a different folded conformation with respect to pose 1 and pose 3 was observed, with a new pi-stacking interaction between the benzyl group and Phe31. The flipped orientations obtained for pose 1 and pose 3 suggested a different entrance mode of the ligand into the active site. Through the BPMD studies, it was possible to observe that both poses were stable, but, according to the RMSD value, the conformation of pose 1 showed major stability compared to pose 3 in the active site. Beyond the van der Waals contacts observed, the conformation of pose 1 could be strengthened by the pi-stacking interactions shown in pose 2 and pose 3 to improve the potency and selectivity of the β1i subunit. To pursue the matter, we also carried out IFD calculations for the other four amide derivatives **2**–**5** that showed an appreciable inhibitory activity on β1i. These studies revealed that Phe31 and Lys33 residues could play a key role in the inhibition pattern, in addition to the already known Ser21 and Gly47 ones, showing the importance not only of the hydrogen bonds but also of the pi-stacking interactions for the stabilization of the binding of the inhibitors. Moreover, the BPMD analysis confirmed the higher binding stability of inhibitor 1 with respect to the inhibitors 2–5, as evidenced by in vitro tests. Compound **1** showed the best CompScore (−1.694) with respect to the other compounds. The consistency of the computational analysis with the experimental data was further confirmed by the MM-GBSA-binding free energy calculations. These outputs were plotted against the experimental *K*i values, and the R^2^ = 0.8071 confirmed compound **1** as the best derivatives of this series. 

## 4. Materials and Methods

### 4.1. System and Ligand Preparation 

For the purposes of this study, we selected the catalytic subunit β1i (LMP2 and PSMB9) extracted from the murine i20S in complex with the inhibitor ONX-0914 bound to the β5i subunit (PDB ID: 3UNF) [20]. Both 20S subunits, murine and human, share a sequence identity of more than 90%, and the few nonidentical residues are external to the active sites. As reported in the literature, in the case of covalent cocrystallized inhibitors [25,26], the reactive residue at the catalytic site was rebuilt after removing the covalent inhibitor by breaking the covalent bond and filling in the open valence. In this case, the involved residue was Thr1. The protein was prepared with the Protein Preparation Wizard [27] included in the Maestro suite (Maestro, Schrödinger, LLC, 2021, New York, NY, USA): adding bond orders and hydrogen atoms to the crystal structure using the OPLS2005 force field. Next, Prime [28] was used to fix missing residues or atoms in the protein and to remove cocrystallized water molecules. The protonation states at pH 7.2 ± 0.2 of the protein and the ligand were evaluated using Epik 3.1 [29]. The hydrogen bonds were optimized through the reorientation of hydroxyl bonds, thiol groups, and amide groups. In the end, the systems were minimized with the value of convergence of the RMSD of 0.3 Å. The ligands were drawn using Marvin Sketch 19.25 [30]. Amide 1 was parameterized using the BiKi suite [19] at the AM1-BCC [22] level of theory. Partial charges were derived using the RESP method [23] in Antechamber [24]. Compounds **2**–**5** were prepared using Schrödinger LigPrep v. 2021-1 (LigPrep, Schrödinger, LLC, 2021, New York, NY, USA). The force field adopted was OPLS2005, and Epik was selected as the ionization tool at pH 7.0 ± 2.0. Tautomers generation was flagged, and the maximum number of conformers generated was set at 32.

### 4.2. MD-Binding Simulations

The MD-binding method [17] within the BiKi suite [19] (BiKi Technologies s.r.l., Genova, Italy) exploits an additive external force that is summed as the regular potential energy of the system to enhance the probability of observing the binding event. The bias is represented by external electrostatic-like forces acting between a subset of the residues of the binding site and the ligand. The intensity of the bias is controlled by the adaptivity rules and gradually switches off as the ligand moves torward the subset of residues; after the conjectured passing of the transition state has occurred, it slowly recovers the behavior of classical unbiased MD [31].

The protocol for MD-binding consists of crucial steps: characterization of the binding pocket using NanoShaper [23] (BiKi Technologies s.r.l., Genova, Italy). NanoShaper calculations provide a characterization of the binding pocket, which identifies the atoms facing the pocket entrance in the protein structure. This information was then used by BiKi software for the initial ligand positioning outside the binding cavity. Subsequently, an additive external force was made to enhance the sampling of the binding event. Once the ligand was positioned through the “Residue Placement” tool in BiKi, the system was solvated in an orthorhombic box using the TIP3P water model [32]. A suitable number of counterions were added to neutralize the overall system. Then, the whole system underwent energy minimization by using the Amber99SB-ildn force field [33]. According to the standard protocol [17], four different consecutive equilibration steps were performed: (1) 100 ps in the NVT ensemble at 100 K with both the protein backbone and ligand restrained (1000 kJ/mol nm^2^), (2) 100 ps in the NVT ensemble at 200 K with both the protein backbone and the ligand restrained, (3) 100 ps in the NVT ensemble at 300 K with the free protein and the ligand restrained, and (4) 1000 ps in the NPT ensemble at 300 K with the free protein and the ligand restrained. Electrostatic interactions were treated with the cutoff method for short-range interactions and with the particle mesh Ewald method for long-range interactions (rlist = 1.1 nm, cutoff distance = 1.1 nm, vdW distance = 1.1 nm, and PME order = 4). The constant temperature conditions were provided using the velocity rescale thermostat [34], which is a modification of Berendsen’s coupling algorithm [35]. The coordinate output from the last simulation was then used as the input to produce the biased molecular dynamics. Finally, 20 replica production runs, 20-ns-long in the NVT ensemble at 300 K, were performed for each complex using C = 0.1 (the fraction of the felt force, here 10%), a smoothing window size of 1000 samples, and a maximal K(t) of 0.001 (maximal steering constant).

### 4.3. Plain MD Simulations 

The plain MD simulations were carried out using Desmond 6.5 [36] using the OPLS2005 force field [37] (Desmond Molecular Dynamics System, D. E. Shaw Research, New York, NY, USA). The complexes were solvated in orthorhombic boxes using the TIP3P water model. Ions were added to neutralize the charges. The systems were minimized and equilibrated at a temperature of 303.15 K and a pressure of 1.013 bar. The system was simulated as an NPT ensemble; a Nose–Hover thermostat and Martyna–Tobia–Klein barostat were used. The integration time step was chosen to be 2 fs. To keep the hydrogen–heavy atom bonds rigid, the SHAKE algorithm was used. A 9 Å cutoff radius was set for the short-range Coulomb interactions, and smooth particle mesh Ewald was used for the long-range interactions. For each replica, we carried out 10-ns MD simulations for a total of 200 ns, with 1.2-ps detection ranges for energy and 4.8 ps for the trajectory frames. The stability of the systems was evaluated using the root mean square deviation (RMSD) of the aligned protein and ligand coordinate set calculated against the initial frame. Visualization and analysis of the MD trajectories were performed using the Desmond simulation analysis tools in Maestro. The trajectories frames were clustered according to the hierarchical cluster linkage method. The 1000 frames recorded in each simulation were clustered considering the binding site conformations into 10 clusters based on the atomic RMSDs. 

### 4.4. Binding Pose MetaDynamics (BPMD)

Binding pose MetaDynamics (BPMD) is an automated, enhanced sampling, metadynamics-based protocol in which the ligand is forced to move around its binding pose. This method showed the ability to reliably discriminate between the correct ligand binding pose and plausible alternatives generated with docking or plain MD studies [18]. 

According to the protocol, 10 independent metadynamics simulations of 10 ns were performed using as a collective variable (CV) the measure of the root mean square deviation (RMSD) of the ligand heavy atoms with respect to their starting positions. The alignment before the RMSD calculations was done by selecting protein residues within 3 Å of the ligand. The Cαs of these binding site residues were then aligned to those of the first frame of the metadynamics trajectory before calculating the heavy atom RMSD to the ligand conformation in the first frame. The hill height and width were set to 0.05 kcal/ mol (about 1/10 of the characteristic thermal energy of the system, kBT) and 0.02 Å, respectively. Before the actual metadynamics run, the system was solvated in a box of SPC water molecules [38], followed by several minimizations and restrained MD steps that allow the system to slowly reach the desired temperature of 300 K, as well as releasing any bad contacts and/or strain from the initial starting structure. The final snapshot of the short unbiased MD simulation of 0.5 ns was then used as the reference for the following metadynamics production phase. After the simulation, the stability of the ligand during the course was represented by three scores: PoseScore, PersistenceScore (PersScore), and CompositeScore (CompScore). The PoseScore is indicative of the average RMSD from the starting pose. A steep increase of this value is a symptom that the ligand is not in a well-defined energy minimum and, probably, it might not have been accurately modeled. PersScore is a measure of the hydrogen bond persistence calculated in the last 2 ns of the simulation that have the same number of hydrogen bonds as the input structure, averaged over all the 10 repeated simulations. It covers a range between 0 and 1, where 0 indicates that either the starting ligand pose did not have any interactions with the target or that the interactions were lost during the simulations, while 1 indicates that the interactions between the staring ligand pose and the last 2 ns of the simulations were retained. CompositeScore is the linear combination of the PoseScore and PersScore; lower values equate to more stable complexes. Each complex, previously obtained, was run, Country) on a single node with a 1 GPU card NVIDIA GeForce RTX2070.

### 4.5. Induced-Fit Docking 

The induced-fit protocol (IFD)—developed by Schrödinger [24]—is a method for modeling the conformational changes induced by ligand binding. This protocol models induced-fit docking of one or more ligands using the following steps, as also reported in references [39,40,41,42]. The protocol starts with an initial docking of each ligand using a softened potential (van der Waals radii scaling). Then, a side-chain prediction within a given distance of any ligand pose (5 Å) is performed. Subsequently, a minimization of the same set of residues and the ligand for each protein/ligand complex pose is performed. After this stage, any receptor structure in each pose reflects an induced fit to the ligand structure and conformation. Finally, the ligand is rigorously docked, using Glide XP (Glide, Schrödinger, LLC, 2021, New York, NY, USA), into the induced-fit receptor structure. IFD was performed using a standard protocol, and the OPLS2005 force field was chosen. The receptor box was centered on the active site of β1i, according to the NanoShaper calculations. During the initial docking procedure, the van der Waals scaling factor was set at 0.5 for both the receptor and ligand. The Prime refinement step was set on the side chains of residues within 5 Å of the ligand. For each ligand docked, a maximum of 20 poses was retained to then be redocked in XP mode.

### 4.6. MM-GBSA-Binding Free Energy Calculations

Prime/MM-GBSA was used for the estimation of ∆G binding. The MM-GBSA approach employs molecular mechanics, the generalized Born model, and the solvent accessibility method to elicit free energies from structural information, circumventing the computational complexity of free energy simulations, wherein the net free energy is treated as a sum of a comprehensive set of individual energy components, each with a physical basis [25]. In our study, the VSGB solvation model was chosen using the OPLS2005 force field with a minimized sampling method [28].

## 5. Conclusions

In this study, we investigated the mechanism of non-covalent inhibition of the potent and selective immunoproteasome inhibitor 1. For this purpose, we employed advanced molecular dynamics methods such as MD binding (MDB) and Binding Pose MetaDynamics (BPMD) and advanced docking methods such as induced-fit docking (IFD). MD binding allowed analyzing the binding mechanisms and gained insights into the ligand entrance pathway. Then, plain MD was performed to study the stability and conformational space in the immunoproteasome–ligand complex, thus allowing elucidation of the compound dynamic behavior within the binding cavity. These results were compared with the IFD poses of the other four inhibitors, revealing new key residues in the binding pattern, and confirmed the different binding stability of **1** with respect to the other compounds, **2**–**5**. The collected information and outcome could be useful in ameliorating the activity of compound 1 and providing a dynamical point of view for the definition of the pharmacophoric features that could be exploited through dynamic pharmacophore modeling approaches, such as the Common Hits Approach (CHA) [43] or MYSHAPE [44], for the scaffold-hopping of new non-covalent inhibitors through a virtual screening campaign.

## Data Availability

Not applicable.

## Supplementary Materials

The following supplementary materials are available online, Figures S1–S12. Figure S1: MD-binding: Ligand RMSD calculated from the centroid of binding pocket and protein backbone RMSD (20 ns) for Replica 1 (**A**,**B**) and Replica 2 (**C**,**D**). Figure S2. MD-binding: Ligand RMSD calculated from the centroid of binding pocket and protein backbone RMSD (20 ns) for Replica 3 (**A**,**B**), Replica 4 (**C**,**D**), Replica 5 (**E**,**F**). Figure S3. MD-binding: Ligand RMSD calculated from the centroid of binding pocket and protein backbone RMSD (20 ns) for Replica 6 (**A**,**B**), Replica 7 (**C**,**D**), Replica 8 (**E**,**F**). Figure S4. MD-binding: Ligand RMSD calculated from the centroid of binding pocket and protein backbone RMSD (20 ns) for Replica 9 (**A**,**B**), Replica 10 (**C**,**D**), Replica 11 (**E**,**F**). Figure S5. MD-binding: Ligand RMSD calculated from the centroid of binding pocket and protein backbone RMSD (20 ns) for Replica 12 (**A**,**B**), Replica 13 (**C**,**D**), Replica 14 (**E**,**F**). Figure S6. MD-binding: Ligand RMSD calculated from the centroid of binding pocket and protein backbone RMSD (20 ns) for Replica 15 (**A**,**B**), Replica 16 (**C**,**D**), Replica 17 (**E**,**F**). Figure S7. MD-binding: Ligand RMSD calculated from the centroid of binding pocket and protein backbone RMSD (20 ns) for Replica 18 (**A**,**B**), Replica 19 (**C**,**D**), Replica 20 (**E**,**F**). Figure S8. Ligand and protein RMSD during MD-plain(10 ns). Replica 1 (**A**); Replica 2 (**B**); Replica 8 (**C**); Replica10 (**D**). Figure S9. Ligand and protein RMSD during MD-plain(10 ns). Replica 11 (**A**); Replica 12 (**B**); Replica 13 (**C**); Replica 14 (**D**). Figure S10. Ligand and protein RMSD during MD-plain(10 ns). Replica 15 (**A**); Replica 16 (**B**); Replica 17 (**C**); Replica 20 (**D**). Figure S11. Ligand Interaction Diagram of pose1 (**A**) and pose2 (**B**). Purple arrows show H-bond interactions and green line Pi-Pi stacking. Figure S12. Ligand Interaction Diagram of pose3 (**A**) and IFD pose (**B**). Purple arrows show H-bond interactions and red line Pi-cation.

## Sample Availability

Samples of the compounds are not available from the authors.
