# Supplementary material for: Immunoproteasome and Non-Covalent Inhibition: Exploration by Advanced Molecular Dynamics and Docking Methods
Source: Molecules. 2021 Jul 2;26(13):4046. doi: 10.3390/molecules26134046 (PMC8271555; doi:10.3390/molecules26134046)
Supplement: Supplementary file 1 [file molecules-26-04046-s001.zip › molecules-1248231-supplementary.pdf]

Supplementary material

Immunoproteasome and non-covalent inhibition: exploration by advanced molecular dynamics and docking methods

Giulia Culletta[1,2], Maria Zappalà[2], Roberta Ettari[2] , Anna Maria Almerico[1], and Marco Tutone[1*]

1 Dipartimento di Scienze e Tecnologie Biologiche Chimiche e Farmaceutiche (STEBICEF), Università degli Studi di Palermo, Via Archirafi 32, 90123 Palermo, Italy;

2 Dipartimento di Scienze Chimiche, Biologiche, Farmaceutiche ed Ambientali, Università di Messina, Viale Annunziata, 98168 Messina, Italy*

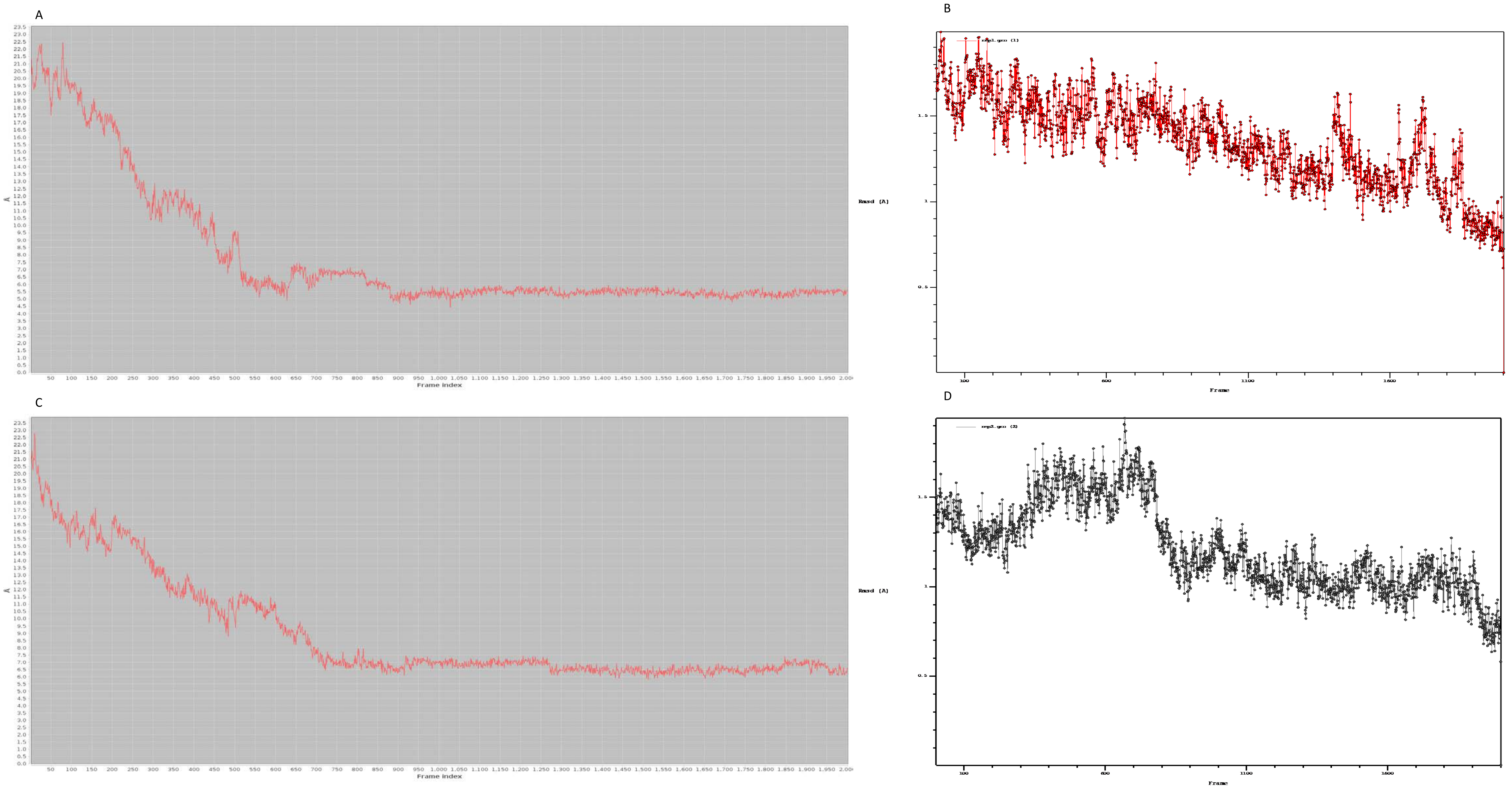

Figure S1. MD-binding: Ligand RMSD calculated from the centroid of binding pocket and protein backbone RMSD (20ns) for Replica 1 (A-B) and Replica 2 (C-D).

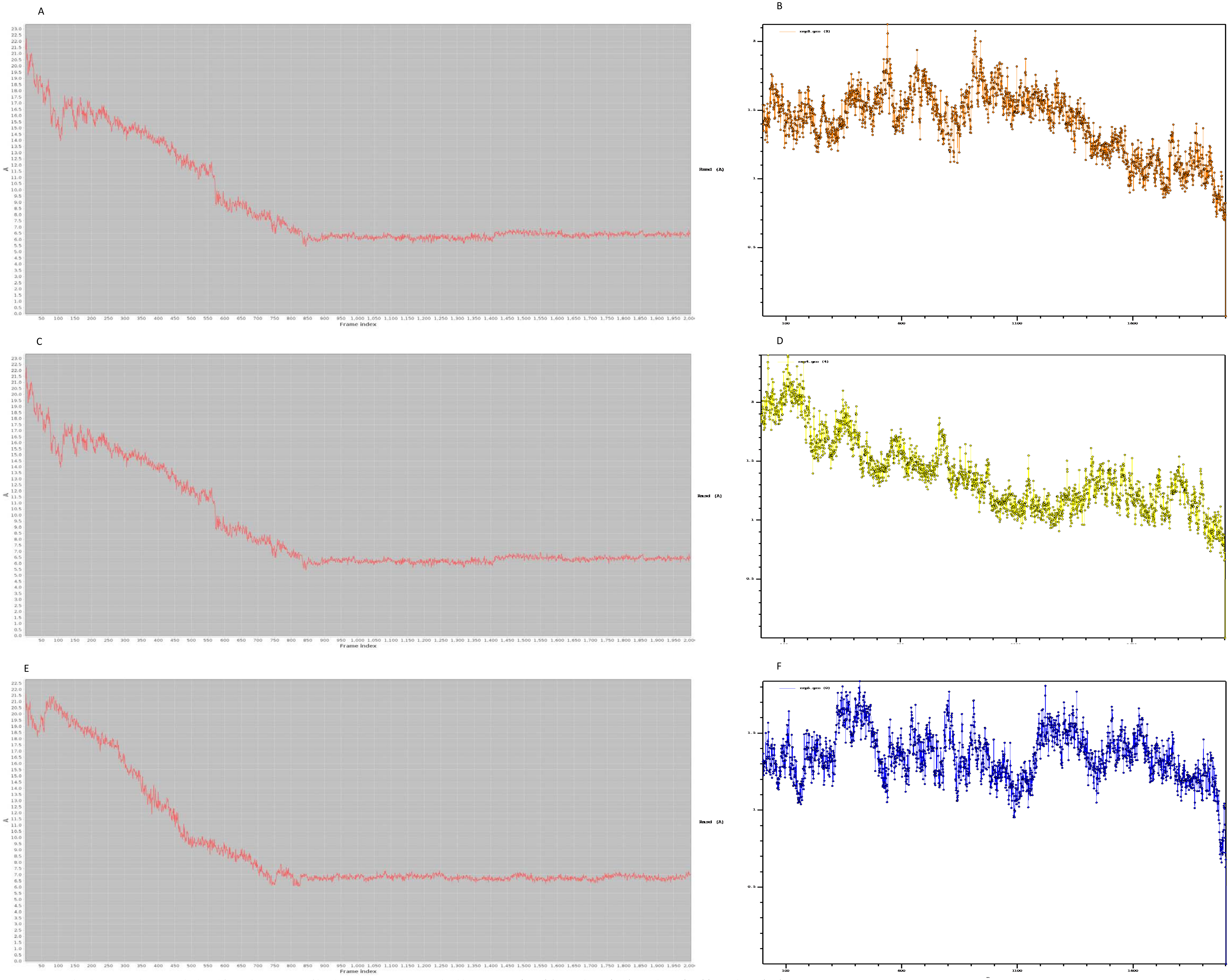

Figure S2. MD-binding: Ligand RMSD calculated from the centroid of binding pocket and protein backbone RMSD (20ns) for Replica 3 (A-B), Replica 4 (C-D)), Replica 5 (E-F).

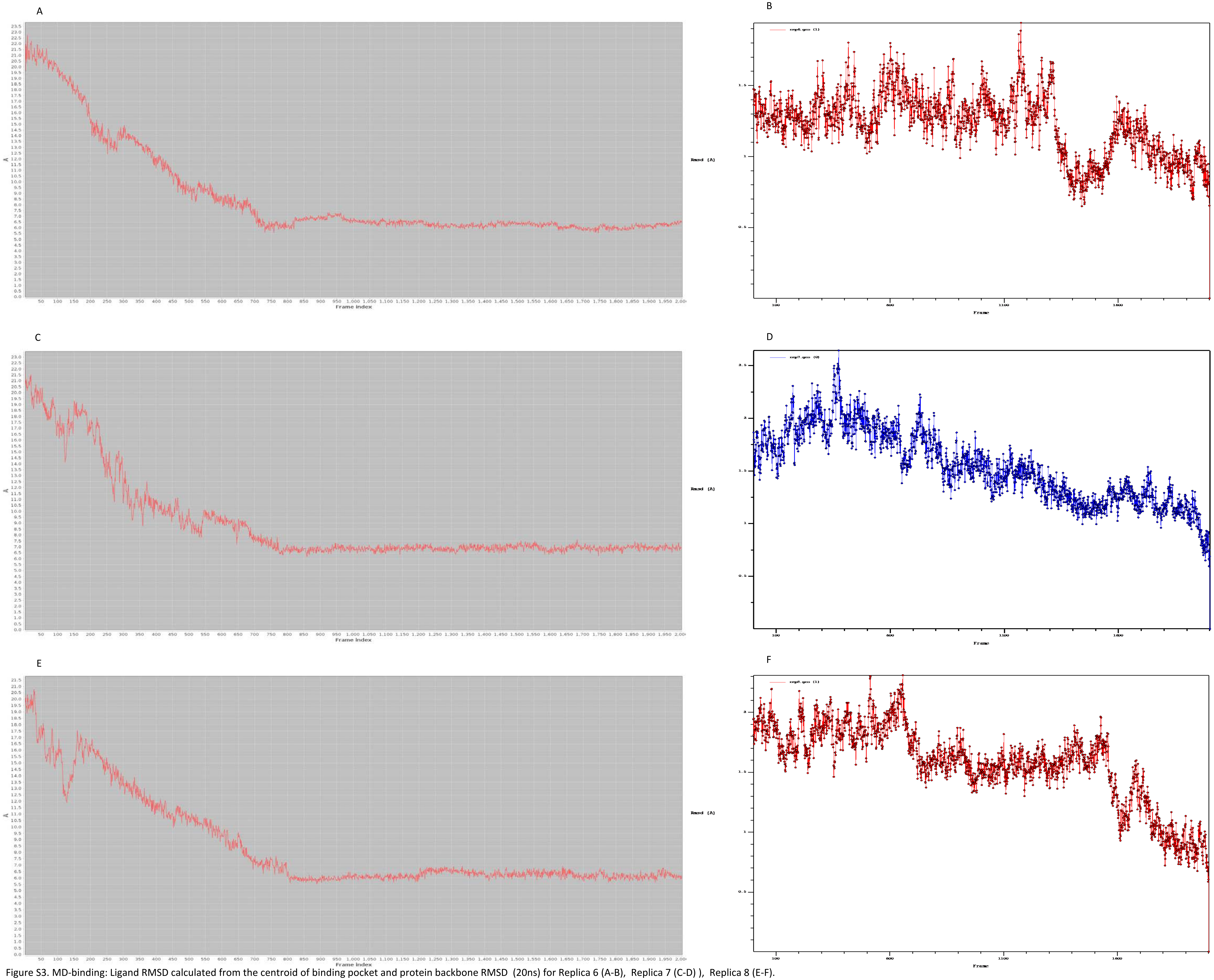

Figure S3. MD-binding: Ligand RMSD calculated from the centroid of binding pocket and protein backbone RMSD (20ns) for Replica 6 (A-B), Replica 7 (C-D)), Replica 8 (E-F).

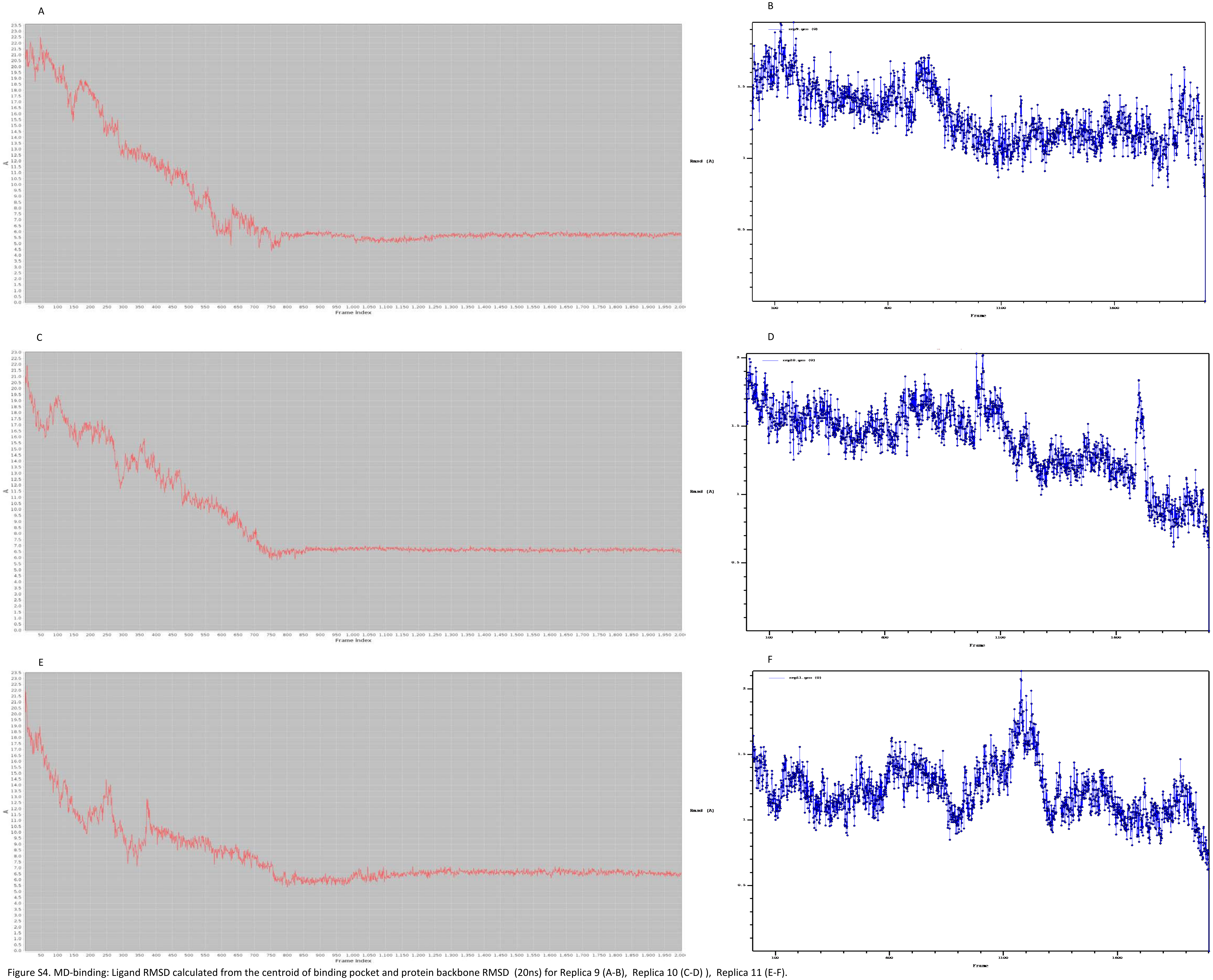

Figure S4. MD-binding: Ligand RMSD calculated from the centroid of binding pocket and protein backbone RMSD (20ns) for Replica 9 (A-B), Replica 10 (C-D)), Replica 11 (E-F).

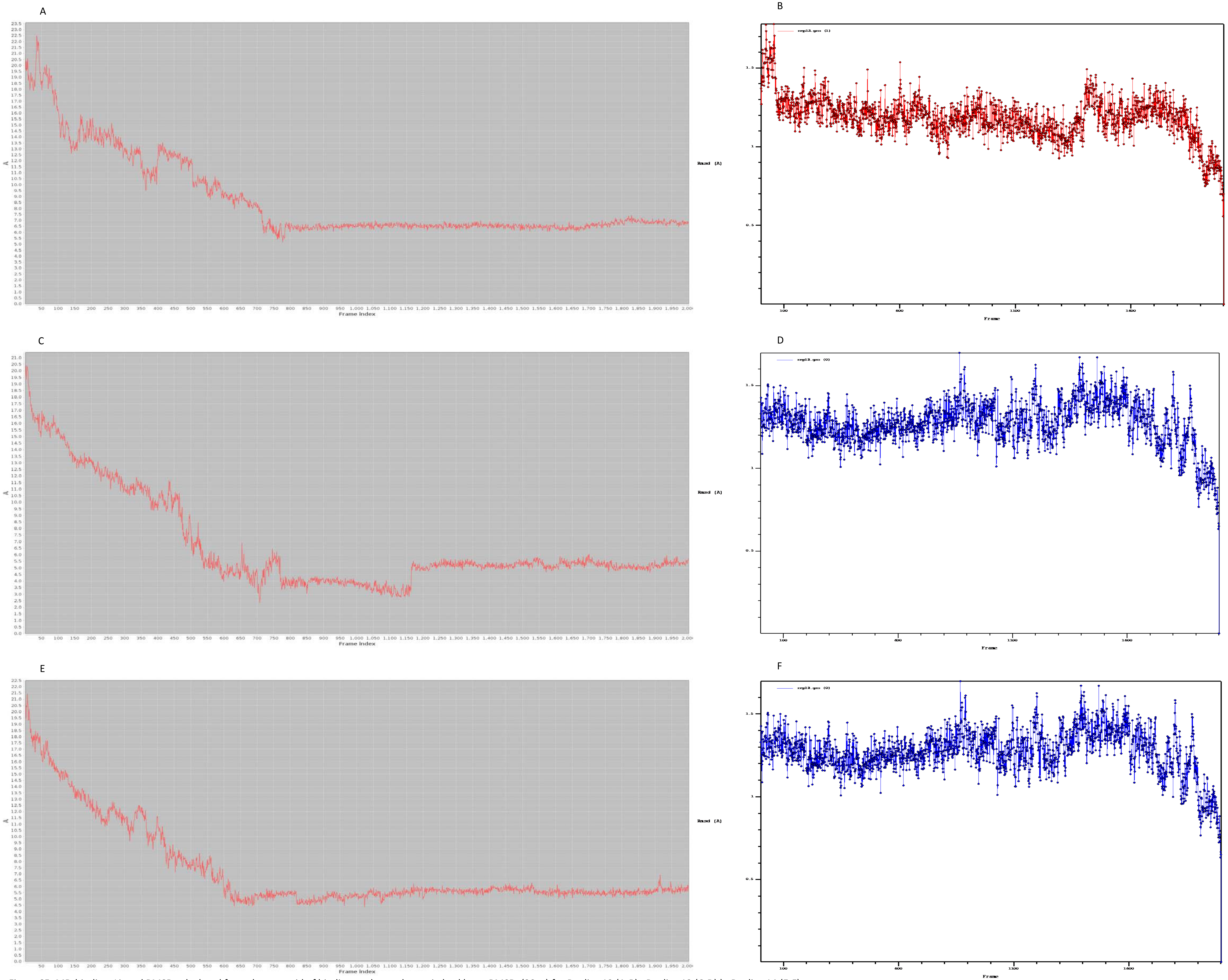

Figure S5. MD-binding: Ligand RMSD calculated from the centroid of binding pocket and protein backbone RMSD (20ns) for Replica 12 (A-B), Replica 13 (C-D)), Replica 14 (E-F).

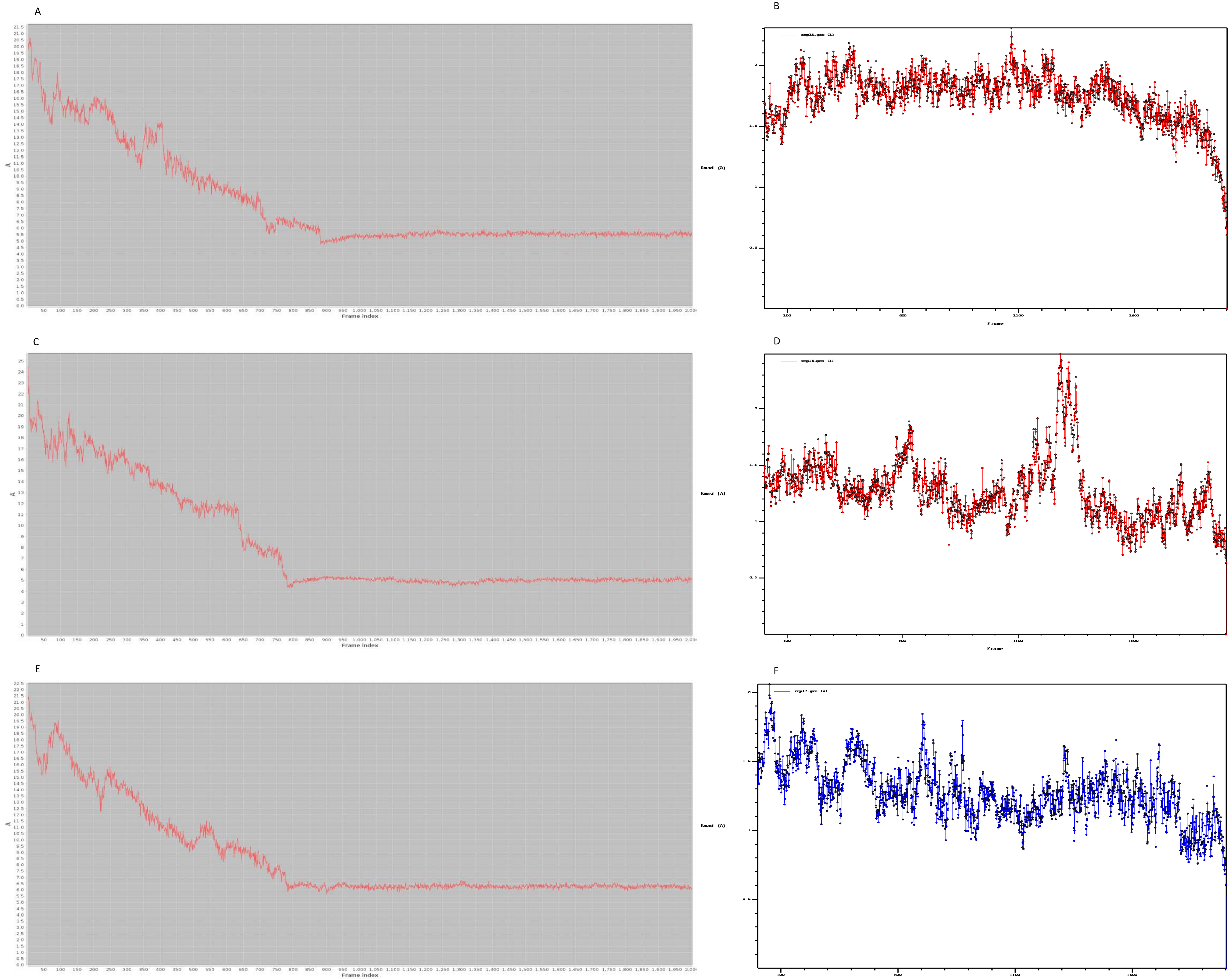

Figure S6. MD-binding: Ligand RMSD calculated from the centroid of binding pocket and protein backbone RMSD (20ns) for Replica 15 (A-B), Replica 16 (C-D)), Replica 17 (E-F).

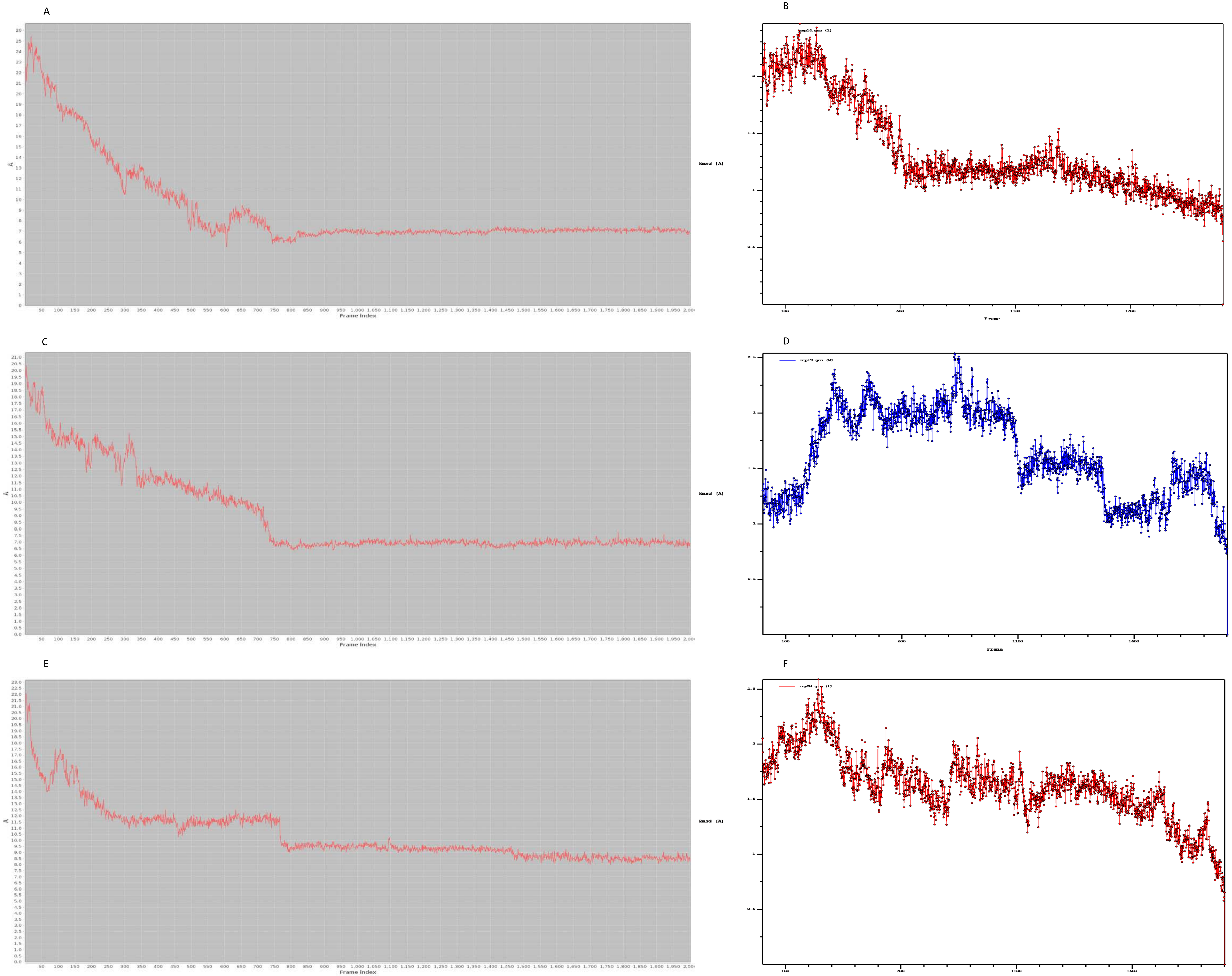

Figure S7. MD-binding: Ligand RMSD calculated from the centroid of binding pocket and protein backbone RMSD (20ns) for Replica 18 (A-B), Replica 19 (C-D)), Replica 20 (E-F).

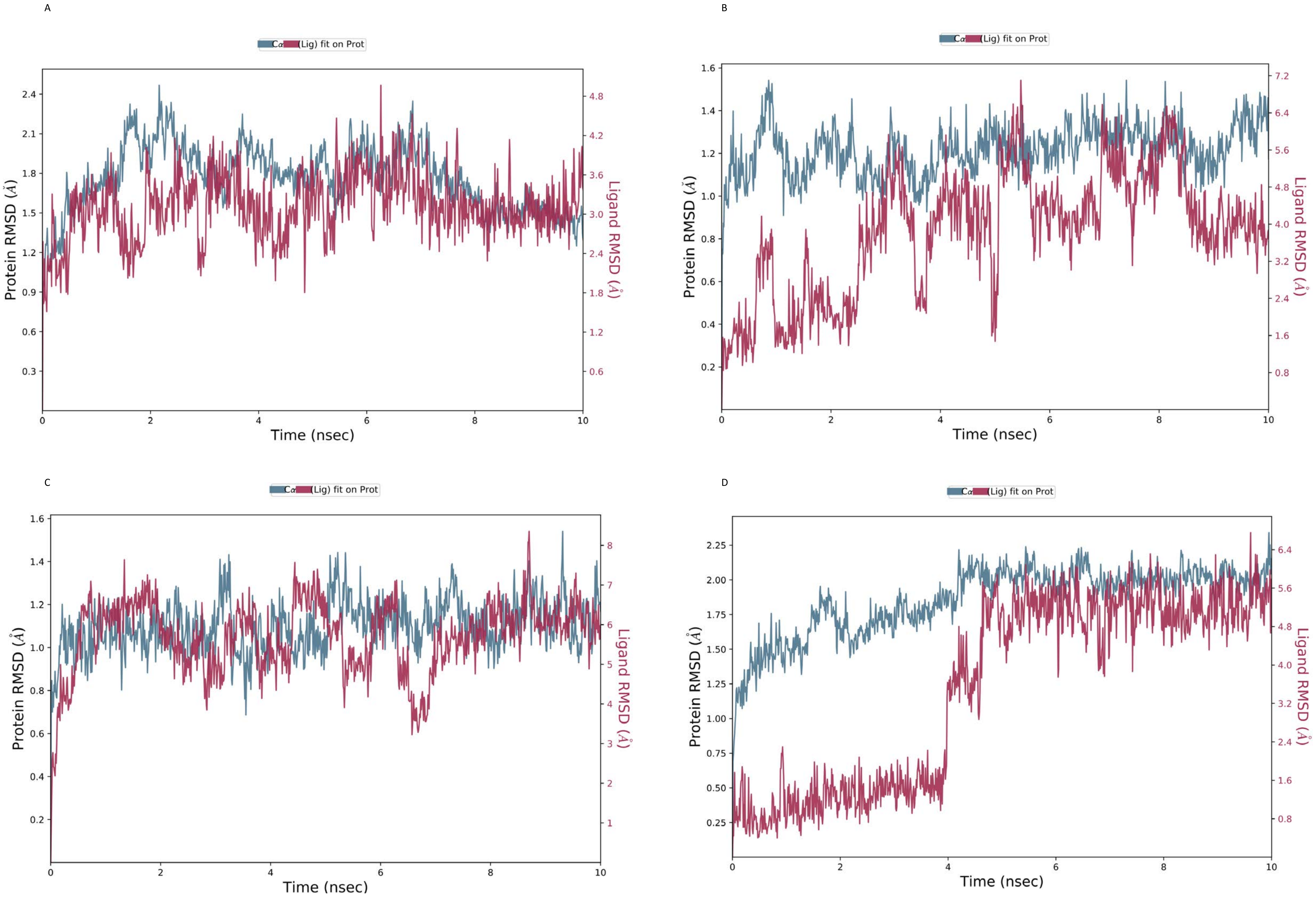

Figure S8. Ligand and protein RMSD during MD-plain(10ns). Replica 1(A); Replica 2(B); Replica 8 (C); Replica10 (D).

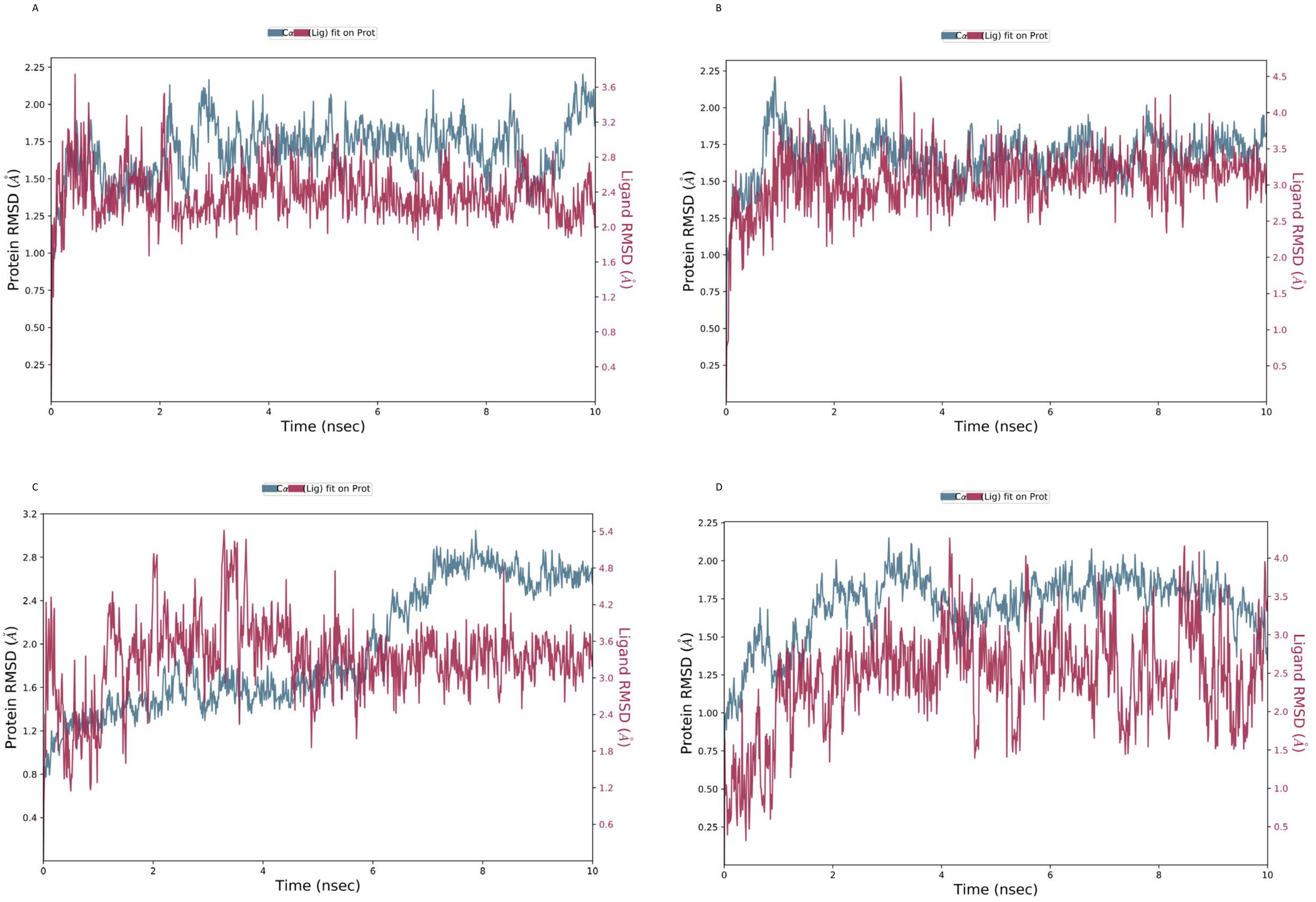

Figure S9. Ligand and protein RMSD during MD-plain(10ns). Replica 11 (A); Replica 12 (B); Replica 13 (C); Replica 14 (D).

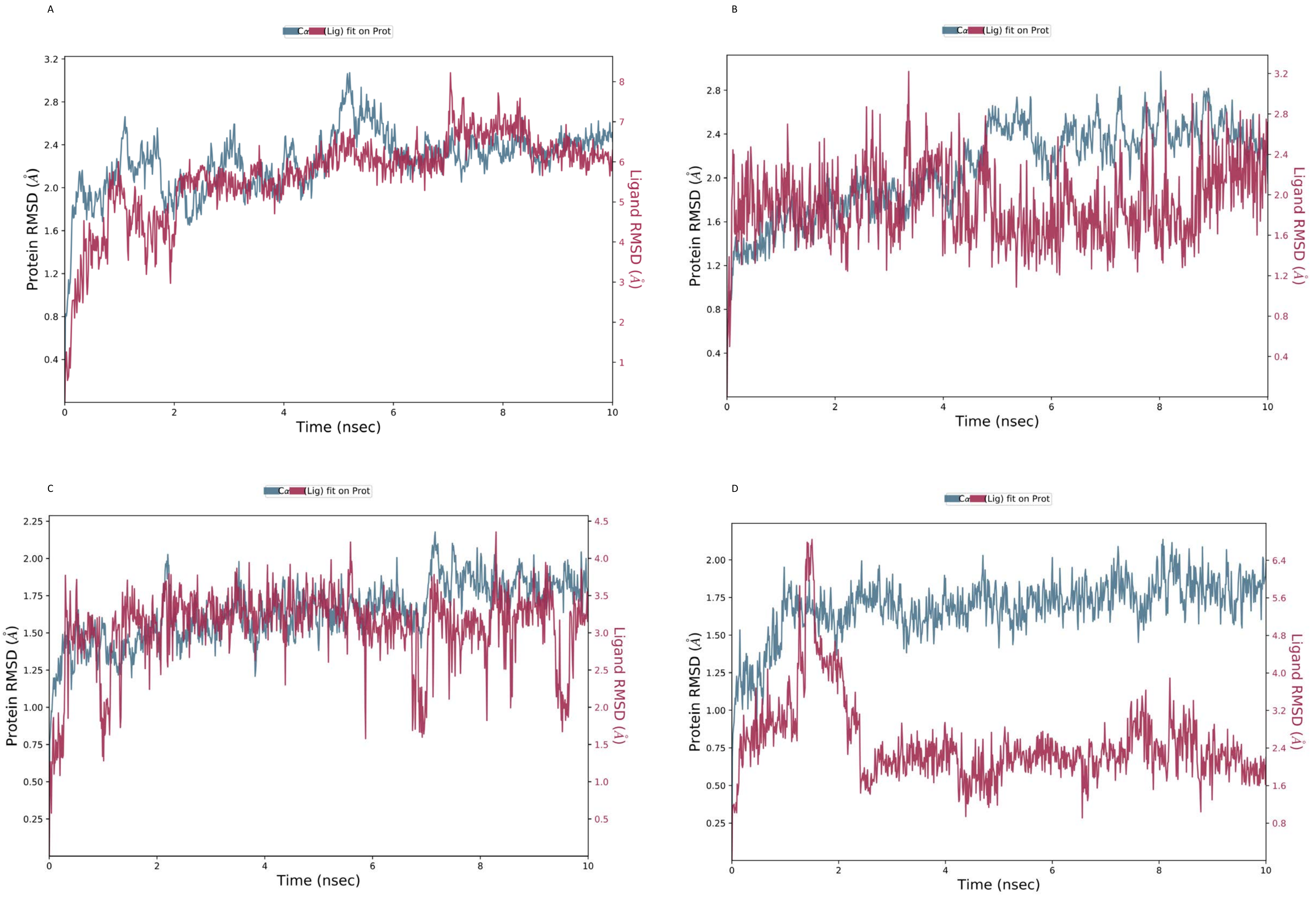

Figure S10. Ligand and protein RMSD during MD-plain(10ns). Replica 15 (A); Replica 16 (B); Replica 17 (C); Replica 20 (D).

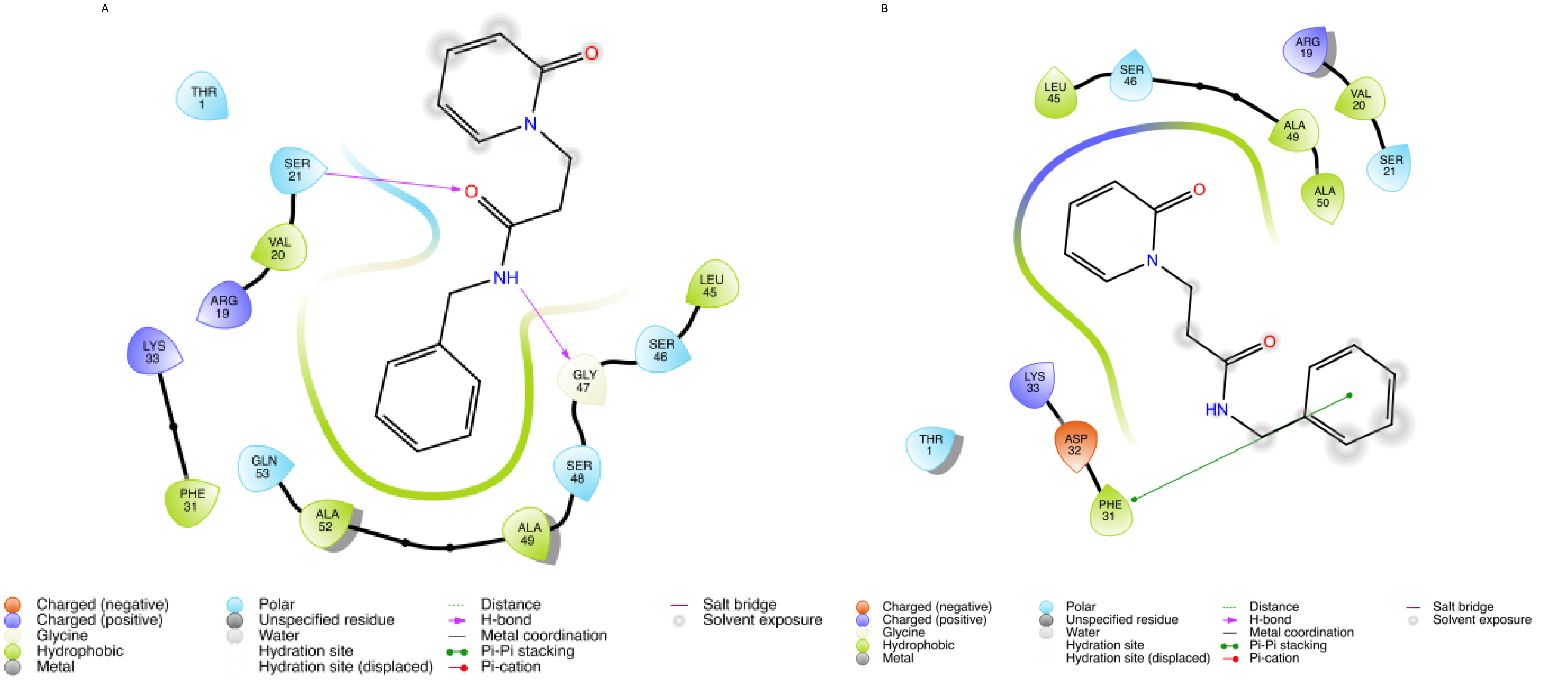

Figure S11. Ligand Interaction Diagram of pose1 (A) and pose2 (B). Purple arrows show H-bond interactions and green line Pi-Pi stacking

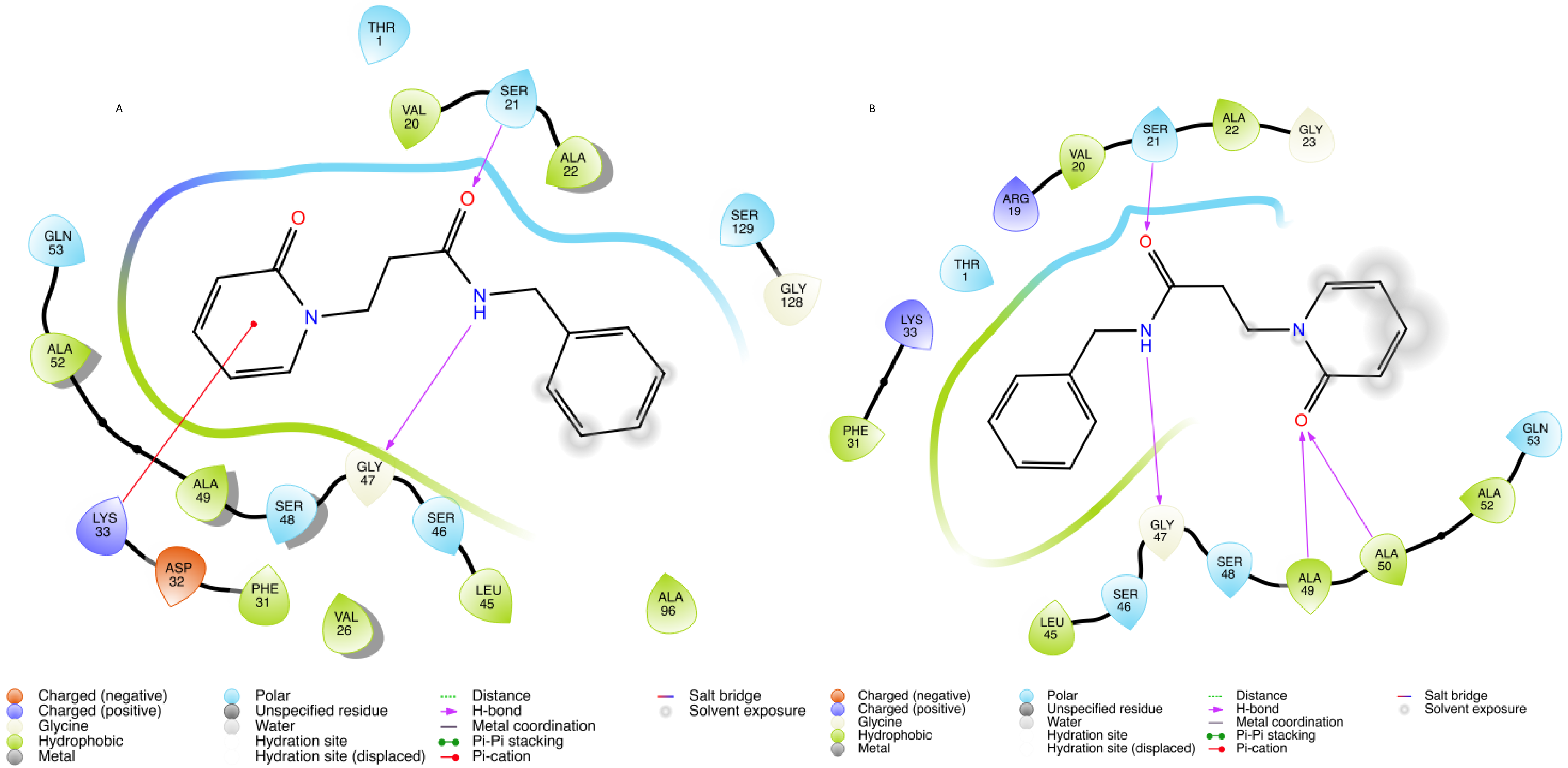

Figure S12. Ligand Interaction Diagram of pose3 (A) and IFD pose (B). Purple arrows show H-bond interactions and red line Pi-cation.
